# Nano Ag/Co_3_O_4_ Catalyzed Rapid Decomposition of *Robinia pseudoacacia* Bark for Production Biofuels and Biochemicals

**DOI:** 10.3390/polym15010114

**Published:** 2022-12-27

**Authors:** Xiaochen Yue, Xiangmeng Chen, Hanyin Li, Shengbo Ge, Yafeng Yang, Wanxi Peng

**Affiliations:** 1 Henan Province Engineering Research Center for Forest Biomass Value-Added Products, School of Forestry, Henan Agricultural University, Zhengzhou 450002, China; 2 Jiangsu Co-Innovation Center of Efficient Processing and Utilization of Forest Resources, International Innovation Center for Forest Chemicals and Materials, College of Materials Science and Engineering, Nanjing Forestry University, Nanjing 210037, China

**Keywords:** *Robinia pseudoacacia* L., TG-FTIR, biofuels and biochemicals, bio-oil, nano Ag/Co_3_O_4_ catalysis

## Abstract

Biomass energy has attracted widespread attention due to its renewable, storage, huge production and clean and pollution-free advantages. Using *Robinia pseudoacacia* bark (RPB) as raw material, biogas and bio-oil produced by pyrolysis of RPB were detected and analyzed by TG-DTG, TG-FTIR and PY-GC-MS under the action of nanocatalysis. TG results showed that CH_4_ and CO flammable gases were produced by pyrolysis. PY-GC-MS results showed that RPB was rapidly pyrolyzed to obtain alcohols, ketones, aldehydes and acids bio-oil. The content of phenolic substances was the highest, accounting for 32.18% of all substances.Nanocatalysis has a certain effect on RPB, accelerating the precipitation of pyrolysis products and improving the over-oxidation of bio-oil. In addition, the extracts of RPB were identified and analyzed by FTIR, NMR, GC-MS and LC-Q-TOF-MS, and more than 100 active ingredients, such as Betaine, Epicathin and β-sitosterol, were detected. Their applications as additive energy in other fields were explored. Therefore, *Robinia pseudoacacia* bark constitutes a fine biofeedstock for biofuels and biochemicals.

## 1. Introduction

The total amount of energy produced and consumed is increasing gradually with the continued development of the economy and the continuous increase in energy consumption. Over-reliance on increasingly depleted fossil energy, especially petroleum energy, has seriously affected the sustainable development of the national economy. Therefore, new, renewable alternative energy sources have become an important way to alleviate energy shortages and reduce environmental pressure. As a new type of energy, biomass energy is renewable and minimally polluting. Biomass energy is in line with the global energy strategy of sustainable development and has great development potential and broad development prospects [1,2,3]. Bio-oil is a mixture of water and complex oxygenated organic matter, that is, a mixture of cellulose, hemicellulose, and lignin degradation products [4,5]. The key technology of biomass oil is fast pyrolysis. This technology theory was put forward in the late 1970s. Crushed crop straw was heated to more than 500 degrees, which prompted a change from pyrolysis of macromolecules to formation of oil vapor, and then to rapid condensation to produce biomass oil [6,7,8].

*Robinia pseudoacacia* L. is a highly adaptable, drought-tolerant plant that is resistant to infertility, easy to reproduce, and that grows fast. The biomass of *Robinia pseudoacacia* is sufficient as a second-generation biomass fuel feedstock [9,10,11,12]. The plant fiber raw material is mainly composed of cellulose, hemicellulose, and lignin. The monomer that composes the cellulose is a hexasaccharide sugar; therefore, it is easily converted into ethanol by a biological or thermochemical method, and hemicellulose can be fermented into ethanol. Lignin cannot be used as a fermentation substrate [13,14]. Studies have shown that *Robinia pseudoacacia* bark (RPB) has relatively high cellulose and hemicellulose contents, but low lignin content, making it suitable as a biomass energy source [15]. In a recent study, seven active ingredients extracted from the root bark of *Robinia pseudoacacia* L., such as medicarpin and liquiritigenin, inhibit the production of ROS and NO in a dose-dependent manner, thus exerting high anti-inflammatory activity [16]. Nanocatalysts can be used to improve the quality of bio-oil by improving the efficiency of pyrolysis reaction and decomposing high-molecular-weight compounds into simpler products [17,18]. Deka et al. found that by adding CeO_2_ nanoparticles, the C–C bond breaking reaction was enhanced, and the percentage of lower molecular weight compounds in the pyrolysis liquid was increased, which significantly improved the pyrolysis process [19]. Ge et al. cracked the used edible oil with impregnated CaO/SBA-15 catalyst in their study to produce liquid hydrocarbons with a high percentage of bio-gasoline fractions [20]. The composite material CaO/SBA-15 effectively cracked 97% of the edible oil into 70% of liquid products and 69.7% of bio-gasoline. In this study, nano-Ag and nano-Co_3_O_4_ were added to *RPB* powder, and pyrolysis-gas chromatography-mass spectrometry (PY-GC-MS) and thermogravimetric-Fourier transform-infrared spectroscopy (TG-FTIR) analyses were carried out to explore the potential of *RPB* as a source of biomass energy.

The *Robinia pseudoacacia* flower is a raw material that can be used to produce honey [21]. In addition to cooling the blood, stopping bleeding, clearing the liver, and purging pathogenic heat, the *Robinia pseudoacacia* flower also clears away heat and detoxifies, cools the blood and moistens the lungs, lowers blood pressure, and prevents stroke [22]. Research on *Robinia pseudoacacia* flowers has been extensive, while research on *RPB* is limited. In this study, the solvent extracts of *RPB* were used as the research object. The main chemical components and contents were analyzed by FT-IR, GC-MS, LC-QTOF-MS and nuclear magnetic resonance (NMR) to determine whether the bark has similar composition and efficacy as the *Robinia pseudoacacia* flower. The results will provide a reference for the development and utilization of *RPB* as a high-quality resource.

## 2. Materials and Methods

### 2.1. Materials

The RPBs were obtained from Xixia County Forest Farm in Henan Province. After freeze-drying, the bark was crushed into powder by a plant crusher. Nanometer silver powder (particle size 60–120 nm, purity of 99.5%) and Cobalt (II, III) oxide powder (particle size 30 nm, purity of 99.5%) were prepared from MACKLIN company.

The first portion was a weighed-out 5.05 g of bark powder. The second portion of 5 g of bark powder was added to with 0.05 g of nano Ag and uniformly mixed. The third portion of 5 g of bark powder was added to with 0.05 g of nano Co_3_O_4_ and uniformly mixed. The fourth portion of 5 g of bark powder was added to with 0.025 g of nano Ag and 0.025 g nano Co_3_O_4_ and uniformly mixed.

**Figure 1:**
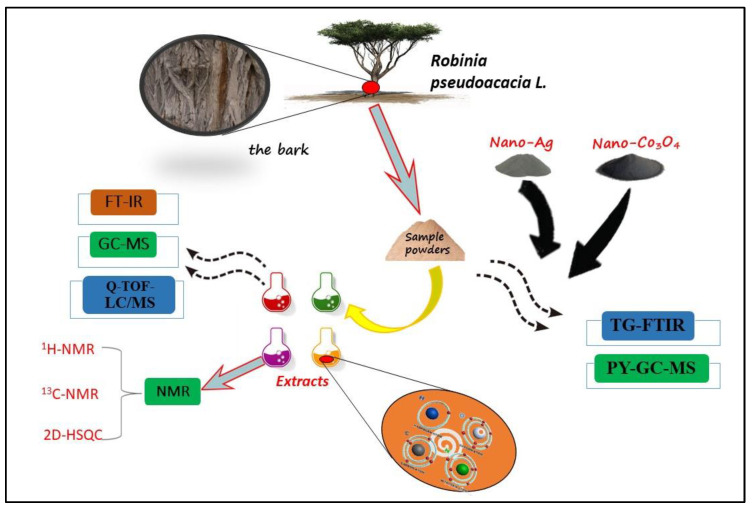
Experimental flow chart of RPB.

The bark powder (30 g/part) was weighed separately. The extract was obtained by an organic solvent extraction method, and then concentrated to about 30 mL by a rotary evaporator to prepare a methanol extract of the bark, a benzene/ethanol extract, and a methanol/ethanol extract. Three copies of the fresh RPB were weighed and crushed into 80–100 mesh, each of about 30 g (0.1 mg accuracy). Extraction was carried out in 300 mL methanol, benzene/ethanol (1:1), and methanol/ethanol (1:1) solvents using the Soxhlet extractor method for 6 extracts at temperatures of 64, 80, and 70 °C, respectively. After extraction, the methanol, benzene/ethanol, and methanol/ethanol were removed via rotary evaporation and dried with anhydrous sodium sulfate, and the resulting extracts were stored at −4 °C for FT-IR, GC-MS, LC-QTOF-MS and NMR detection as seen in Figure 1.

### 2.2. Experimental Methods

***TG-DTG experiments***

Through TG (TGA-Q50, TA Instruments, New Castle, DE, USA) analysis, under the control of program temperature, the change of the real-time weight-temperature relationship is shown. It can be used to identify the thermal stability of samples and analyze the decomposition process and pyrolysis mechanism of substances. The temperature program started at 30 °C and was increased to 850 °C at the heating rate of 20 °C/min [23,24].

***Thermogravimetric–Fourier-Transform-Infrared (TG-FT-IR) experiments***

To analyze volatile gas components or decomposition products produced during weightlessness, the TG-FT-IR analysis of samples was performed using a TG analyzer (TGA Q500, TA Instruments, New Castle, DE, USA) connected to a Fourier-transform infrared spectrometer. The temperature was increased from 50 to 950 °C at a rate of 60 °C/min. Three-dimensional (3D) FT-IR spectrograms were obtained after the experiments by the Thermo Scientific Nicolet™ 6700 FT-IR spectrometer [25,26].

***Pyrolysis-gas chromatography-mass (PY-GC-MS) analysis***

The four powdered samples were analyzed by thermal cracking-gas chromatography-mass spectrometry (CDS 5000-Agilent 7890B-5977A, Agilent Technologies, Palo Alto, CA, USA). The pyrolysis temperature was raised to 950 °C at the rate of 20 °C/ms, and the retention time was 15 s. The specification of the capillary column was 30 m × 0.25 mm × 0.25 μm (TR-5MS column). The split ratio is 50:1, and the temperature of GC program starts from 40 °C, lasts for 2 min, increases to 120 °C at the rate of 5 °C/min, and then increases to 200 °C at the rate of 10 °C/min, lasts for 15 min [27,28].

***FT-IR analysis***

The infrared spectrum data of the extracted samples were obtained using an FT-IR spectrophotometer (iS10, Thermo Fisher Scientific, Waltham, MA, USA), containing a 1.00% finely ground sample.

***NMR analysis***

An NMR polarimeter (Agilent-400 MR; Agilent Technologies, Palo Alto, CA, USA) was used with methanol-d4 as the solvent. One NMR probe was used to determine ^1^H-NMR, ^13^C-NMR, and two-dimensional (2D)-NMR. ^1^H-NMR: duration, 1.000 s; pulse, 45°; sample-and-hold time, 2.556 s; pulse width, 6410.3 Hz. ^13^C-NMR: duration, 1.000 s; pulse, 45°; sample and hold time, 1.311 s; pulse width, 25,000.0 Hz. 2D-Heteronuclear single-quantum correlation (HSQC): duration, 1.000 s; sample and hold time, 0.150 s; two pulse widths of 4807.7 Hz and 20,105.6 Hz.

***GC-MS analysis***

GC/MS determination: The extracts was performed on a GC-MS (Agilent 7890B-5977A; Agilent Technologies, Palo Alto, CA, USA). The column employed was a HP-5MS column (250 μm × 0.25 μm × 30 m). Flow rate was set at 1 mL/min, and the split ratio was 2:1. The GC temperature program started at 50 °C and was increased to 250 °C at a rate of 8 °C/min, and then to 280 °C at a rate of 5 °C/min. A scan mass range program of 30–600 amu was used, and the ion source and the quadrupole temperatures were set to 230 °C and 150 °C, respectively [28,29].

***LC-QTOF-MS analysis***

The ethanol/methanol extracts were analyzed by the Agilent 1260/1290 HPLC + 6530/6545/6550 QTOF detector. The following LC and MS parameters were provided by Agilent Co. (Beijing, China). LC: The chromatographic column was Agilent Eclipse Plus C18 (2.1 × 100 mm, 1.8 μm). Mobile phase–positive ion mode: 0.10% (*v*/*v*) formic acid (A), acetonitrile with 0.10% (*v*/*v*) formic acid (B). MS: Ion source: AJS ESI. Detection mode: Positive ion mode. Scan mass range program: 50–1200 *m*/*z*.

## 3. Results and Discussion

### 3.1. Behavior during Combustion of RPB via Catalysis

#### 3.1.1. TG Analysis

**Figure 2:**
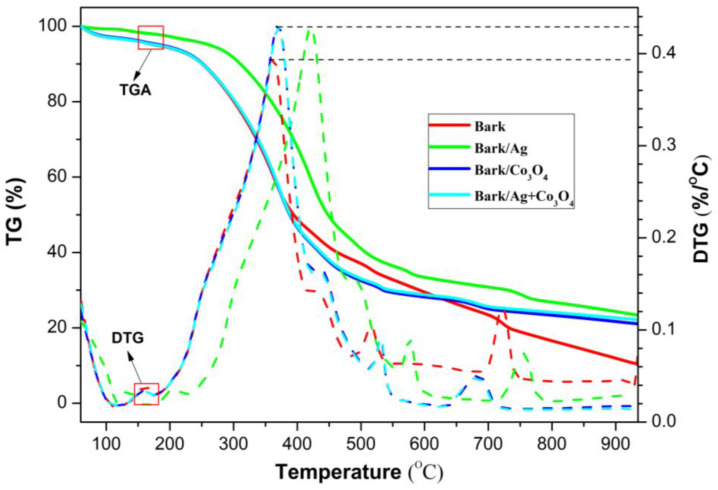
TG and DTG curves of the RPB samples.

Figure 2 shows the TG/derivative thermogravimetry (DTG) plot of the original bark and the bark with different catalysts added. According to the results in Figure 2, the weight loss process is roughly divided into three stages. The first stage is from the initial temperature to 200 °C. This stage included the micro-weight loss process, mainly due to the evaporation of some water and the removal of a small amount of volatile compounds. The bark samples, bark/Co_3_O_4_ samples, and bark/Ag-Co_3_O_4_ samples had a weight loss rate of 5.8% at this stage, and the bark/Ag samples weight loss rate was 3.1%.

The second stage was 200–600 °C. The weight loss from this process was much more significant. The weight loss rate of bark samples, bark/Co_3_O_4_ samples, and bark/Ag-Co_3_O_4_ samples was 71.3% at this stage, and the weight loss rate of bark/Ag samples was 66.4%. The weight loss from this process may have been due to decomposition of cellulose, hemicellulose, and lignin [30,31].

The final stage was 600–950 °C. The weight loss was slower, the weight loss rate was relatively small, and the final curve tended to be flat. The final residual content of bark samples was 10.45% of the initial content. The final residual of bark/Ag samples was 23.41% of the initial content, and the final residual of bark/Co_3_O_4_ samples was 21.09% of the initial content. Bark/Ag-Co_3_O_4_ samples were 22.18% of the initial content. The remaining amount of bark samples were the least, and the residual amount of sample added to with nanocatalyst was relatively large, indicating that nano-Ag and nano-Co_3_O_4_ had no obvious catalytic effect on the total weight of the bark during pyrolysis of the biomass. Non-cracking of the nano-metal powder resulted in more residual than the original bark powder.

The DTG curve peaked at a temperature of about 400 °C. The fastest pyrolysis rate of cellulose and hemicellulose occurred at this temperature. The formation of bio-oil made a significant contribution. The rate began to decrease gradually as temperature was increased. The maximum peak value of DTG of the bark/Ag sample (0.428%/°C), bark/Co_3_O_4_ sample (0.431%/°C), and bark/Ag-Co_3_O_4_ samples (0.429%/°C) peaks with the metal nanocatalysts were larger than the original bark sample powder (0.394%/°C), indicating that the metal catalyst increased the rate of pyrolysis.

#### 3.1.2. TG-FTIR Analysis

**Figure 3:**
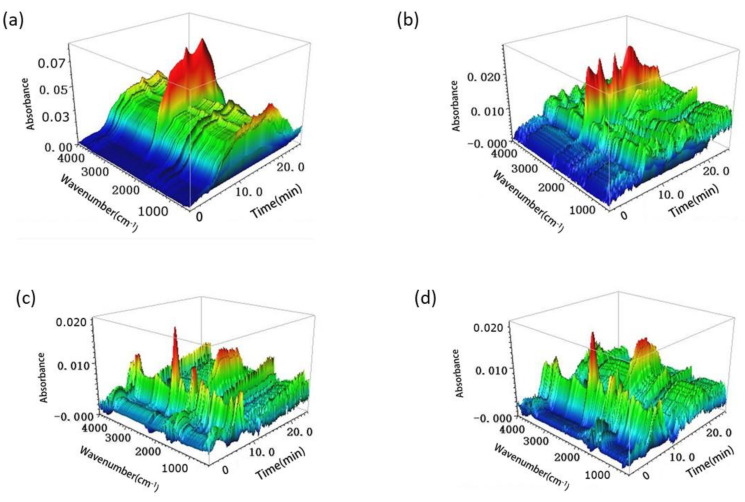
3D FTIR spectrograms of bark (**a**), bark/Ag (**b**), bark/Co_3_O_4_ (**c**), and bark/Ag-Co_3_O_4_ (**d**).

The structure of the 3D-FT-IR spectrum of the pyrolysis volatiles is shown in Figure 3 for the bark samples, bark/Ag samples, bark/Co_3_O_4_ samples and bark/Ag-Co_3_O_4_ samples. TG-FT-IR technology analyzes the mass change characteristics of the sample pyrolysis gasification process and quickly analyzes the formation and release characteristics of the gaseous products [32,33]. The first 5 min of pyrolysis creates the product precipitation spectrum during the initial stage of pyrolysis. It can be clearly observed that the first 5 min was characterized by an H_2_O gas peak at 3800–3500 cm^−1^. This process is mainly caused by evaporation of residual moisture in the sample. The sample had the highest thermal absorbance during the main stage (5–20 min) of pyrolysis. The IR light absorption characteristic frequency of the CO_2_ gaseous product was 2400–2200 cm^−1^, that of CH_4_ was 3200–2700 cm^−1^, and that of the carbonyl groups (C=O) was 1900–1650 cm^−1^. At the same time, strong absorption peaks appeared in the ranges of 3000–2650 cm^−1^, 1850–1600 cm^−1^, and 1500–1000 cm^−1^, indicating the stretching vibrations of CH bonds, C=O double bond stretching vibrations, and CH key in-plane bending vibrations, respectively. These absorption peaks correspond to macromolecules, such as hydrocarbons, aldehydes, alcohols, and acids [34,35]. As the temperature continued to rise, the thermal decomposition stage of the pyrolysis residue occurred after 20 min, at which stage CO precipitates as the main gas product. The C-H and C-O bonds are further cleaved and undergo conversion by aromatization.

Based on the above analysis, the pyrolysis gasification process of bio-oil was mainly divided into two stages, such as volatilization pyrolysis of the light components under low-temperature conditions and pyrolysis gasification and condensation coking of the heavy components under high-temperature conditions. Comparing the four images, the pyrolysis start time of adding nano metal materials (3 min) to the bark sample was earlier than that of the original bark powder (5 min), and the pyrolysis takes place more fully; more pyrolytic gas absorption peaks were generated in 5–20 min and over 20 min.

#### 3.1.3. PY-GC-MS Analysis

**Figure 4:**
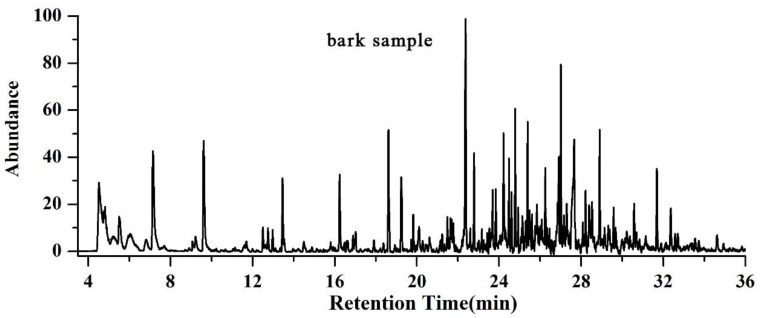

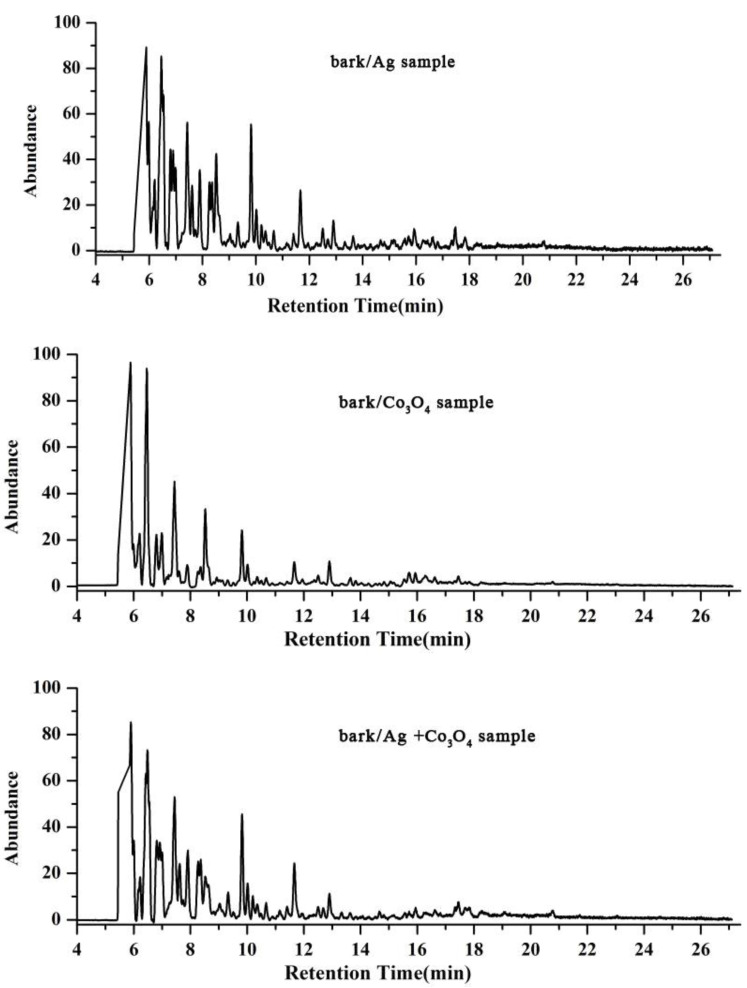
Total ion chromatograms of RPBs by PY-GC-MS.

The total ion chromatograms of RPBs from PY-GC-MS were shown in Figure 4. The relative content of each component was determined by area normalization, and specific results were shown in Tables S1–S4. The MS data were analyzed using the standard NIST MS map to identify each component [36].

According to the results for bark sample, 156 peaks were detected. The results show the following percentages of contents: catechol (6.84%), benzene (5.64%), toluene (4.74%), trans-isoeugenol (3.48%), 1-propynyl-benzene (3.46%), 2-methoxy-4-vinylphenol (2.82%), 3-methyl-phenol (2.53%), 2,6-dimethoxy-phenol (2.52%), 4-methyl-1,2-benzenediol (2.48%), 2,3,5,6-tetrafluoroanisole (2.38%), phenol (2.23%), 2,3-dihydro-benzofuran (2.14%), indole (1.99%), styrene (1.97%), cyclopropylacetylene (1.83%), 3-methoxy-1,2-benzenediol (1.47%), 2-ethyl-1,1’-biphenyl (1.58%) and 2-methoxy-phenol (0.83%).

According to the analysis for bark/Ag sample, 104 peaks were detected. The results show the following percentages of contents: 2-butene (16.74%), acetone (9.93%), acetic acid ethenyl ester (6.44%), acetic acid (4.66%), toluene (4.40%), 4-methyl-1-pentene (3.61%), 2-methyl-propanal (3.27%), acetonitrile (3.14%), acetaldehyde (3.09%), benzene (3.04%), furan (2.75%), 1-heptene (2.11%), pyrrole (2.58%), furfural (1.13%), and 2,3-butanedione (0.53%).

According to the results for bark/Co_3_O_4_ sample, 76 peaks were detected. The results show the following percentages of contents: 2-butene (19.22%), acetone (14.59%), acetaldehyde (3.74%), cyclopropane, 1,1-dimethyl-(1.17%), furan (2.64%), methyl isocyanide (3.05%), 2-methyl-propanal (3.85%), guanidine (6.12%), 1-heptene (2.31%), benzene (2.96%), 3-methyl-butanal (4.52%), acetic acid (1.80%), toluene (4.22%), 1-methyl-1H-pyrrole (1.26%), pyrrole (2.52%), furfural (1.0%), 2-methoxy-4-vinylphenol (0.86%), and 5H-1-pyrindine (0.79%).

According to the results for bark/Ag + Co_3_O_4_ sample, 85 peaks were detected. The results show the following percentages of contents: acetone (15.76%), 2-butene (14.46%), acetic acid ethenyl ester (6.85%), 3-methyl-butanal (4.76%), pyrrole toluene (4.50%),methyl isocyanide (4.09%), benzene (3.37%), 2-methyl-propanal (3.13%), pyrrole (2.97%), acetaldehyde (2.93%), furan (2.62%), 1-heptene (2.48%), 2-methyl-furan (2.26%), acetic acid (2.20%), 1-methyl-1H-pyrrole (1.52%), (E,E)-2,4-hexadienal (1.35%), furfural (1.24%), 2-octene (1.14%), 1,1-dimethyl-cyclopropane (0.97%) and creosol (0.48%).

After rapid pyrolysis, large amounts of small molecular compounds which can be used to prepare bio-oil were obtained. Each bio-oil has its own unique components, but the main components belong to the same chemical group, including organic acids, alcohols, ketones, aldehydes and phenols. Among them, the content of phenolic substances was the highest, accounting for 32.18% of all substances. The phenols detected were catechol, trans-isoeugenol, 3-methyl-phenol, phenol, creosol and so on. Phenols are widely used as raw materials for many chemicals. In addition, studies at the University of California (USA) have shown that red wine contains phenolic compounds with higher antioxidant capacity than vitamin E and can effectively reduce artery stenosis caused by infarction. Ketones and a small amount of aldehydes in bio-oil make bio-oil hydrophilic, which is also the reason why it is difficult to remove water from bio-oil. The acetone content is the highest in ketones, and aldehydes contain 2-methyl-propanal, acetaldehyde, furfural, etc. Formic acid and acetic acid are higher in the acids produced by bark cracking.

Nanocatalysis has a certain effect on biomass oil. From the point of view of the precipitation of pyrolysis products, nanocatalysts can accelerate the precipitation of products. High oxygen content is the main obstacle to the application of bio-oil. Bio-oil can be prepared by rapid pyrolysis of biomass under the action of nanocatalysis, which can reduce the oxygen content of bio-oil, thereby improving the calorific value and stability of bio-oil [20,37,38].

### 3.2. Extractives of RPB for Added Energy

Rapid pyrolysis of biomass yields fewer bio-oil components, and the cost of separation and refining is higher. Therefore, it is necessary to extract other high value-added chemicals from biomass energy and apply them to other fields. Plant extracts refer to the substances extracted from plant products by using organic solvents such as ethanol, acetone, methanol, benzene or petroleum ether and aqueous solution [39,40]. By studying the components of plant extracts, we can not only understand the color, aroma and durability of plants more directly, but also put forward reasonable suggestions for plant processing and utilization. In addition, the active ingredients obtained from plant extracts also play an important part in industry, the food industry and the pharmaceutical industry. FTIR, NMR, GC-MS and LC-QTOF-MS were used to identify and analyze the active components of RPB extractives, so as to maximize the additional energy of biomass energy.

#### 3.2.1. FT-IR Analysis

**Figure 5:**
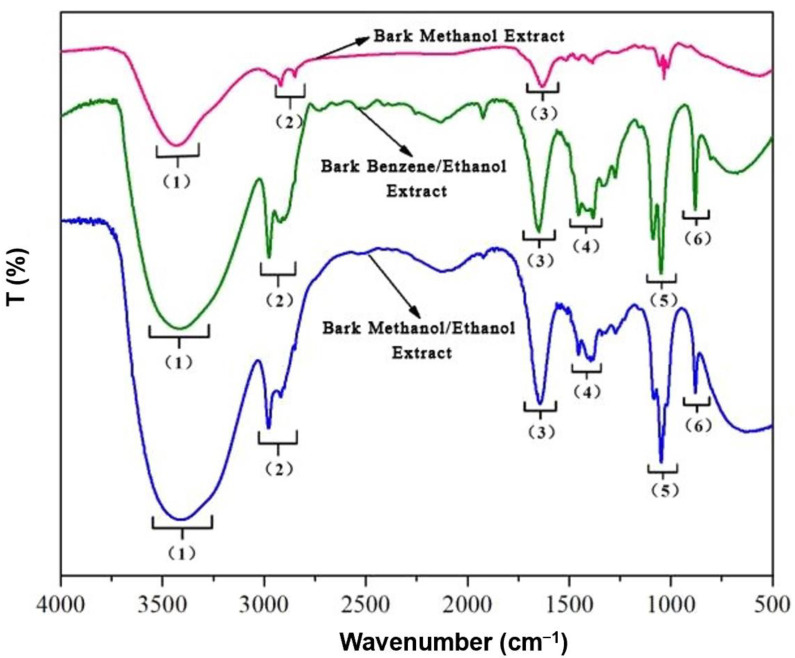
The FT-IR spectra of the RPB extracts.

The IR spectrum of RPB was analyzed according to the relationship between the IR spectrum of the organic compounds and the functional groups. The IR contrast spectra of RPB extracts was shown in Figure 5 and Table 1.

The absorption peaks of the extracts were mainly concentrated in the 3730–3000 cm^−1^, 1750–1550 cm^−1^, and 1100–850 cm^−1^ bands. Absorption peaks were formed by shaking or anti-stretching vibrations of free hydroxyl groups in liquid water in the 3400 cm^−1^ or higher IR spectrum. The appearance of the absorption peak at 3400–3300 cm^−1^ was formed by NH_2_ symmetric and antisymmetric stretching. Furthermore, the appearance of the absorption peak at 3030 cm^−1^ represented stretching vibrations of -CH bonds. These two peaks indicate the presence of phenols and alcohols in the extract. The absorption peaks that appeared at 1730–1550 cm^−1^ were caused by stretching vibrations of C=O double bonds. This position indicates the presence of esters, ketones, and acids in the extract. The absorption peak appearing in the 1100–850 cm^−1^ band was caused by NO_3_^−^ symmetric stretching, and the absorption peak at 900 cm^−1^ was formed by bending COH out-of-plane. The cellulose absorption peak (3500–3300 cm^−1^) decreased significantly, indicating hydrolysis of cellulose [41]. The hemicellulose (3400 and 2970 cm^−1^) and lignin (1470–1430 cm^−1^) absorption peaks decreased slightly, indicating that hemicellulose and lignin were less hydrolyzed than cellulose [42,43]. Comparing the three graphs at the absorption peak of 3400 cm^−1^, the absorbance of the bark samples-1 methanol extract decreased less, while the absorbance of the bark samples-2 benzene/ethanol extract and bark samples-3 methanol/ethanol extract decreased significantly, indicating that ethanol is a more suitable extraction solvent to extract phenolic substances. Phenolic compounds are the main component affecting the color of wood, which explains why wood is lighter during organic solvent extraction.

#### 3.2.2. Analysis of NMR Spectra

**Figure 6:**
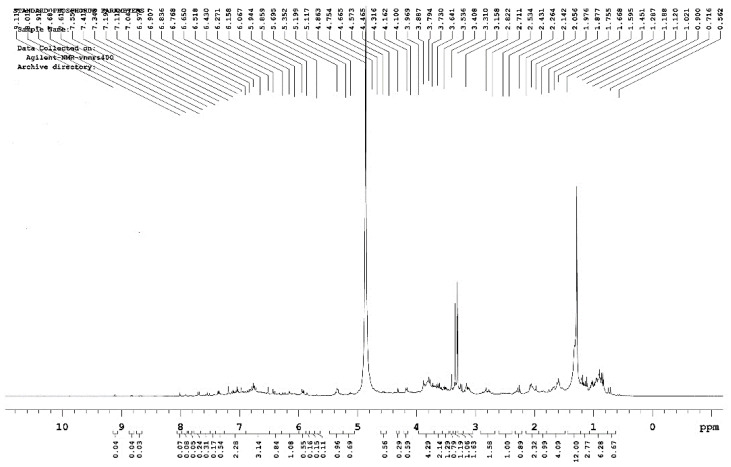
^1^H-NMR spectra of RPB sample.

The ^1^H-NMR spectrum is the most widely used NMR spectrum. The hydrogen nucleus has a large magnetic spin ratio γ, resulting in strong magnetic properties, high detection sensitivity, and easy-to-observe signals. A large number of hydrogen atoms are found in the different chemical environments of organic compounds, so the ^1^H-NMR spectrum provides important structural information for many organic compounds [44,45]. The H spectrum (Figure 6) revealed that the chemical shifts in protons mainly occurred at the 1.3, 3.3, and 4.8 ppm positions. The 1.3 ppm δ value position represents the chemical shift value for the alkane proton-C-C-H. This position may represent the CH_3_OH solvent peak, as it is near the delta value of 3.3–3.4 ppm. The chemical shift of the proton on the carbon atom directly connected to a halogen is also present in this position. A small peak appeared at the 5.3 ppm position, which may be the chemical position value for R_2_C=CRH protons. The δ value increased when coupled with an aryl group, particularly at δ 5.3 and δ 6.8 ppm, where the peak was most pronounced. The peak appearing near the δ value of 6.7 ppm may be the chemical shift that occurs with the unsubstituted aromatic ring of an aromatic proton. The chemical shift in the aromatic compounds ranged from 6.3 to 8.5, and the value of the heterocyclic aromatic proton was in the range of 6.0 to 9.0. The chemical shift in H on the aldehyde group generally occurs at the 9–10.5 ppm position. The H chemical shift of the alcohol species was present at the 0.5–5.5 ppm position, while the H chemical shift of the phenolic species generally occurs at the 4.0–8.0 ppm position [46,47,48]. The predominant compounds in RPB can be roughly determined according to the peaks of the specific chemical shifts shown by the ^1^H-NMR spectrum.

**Figure 7:**
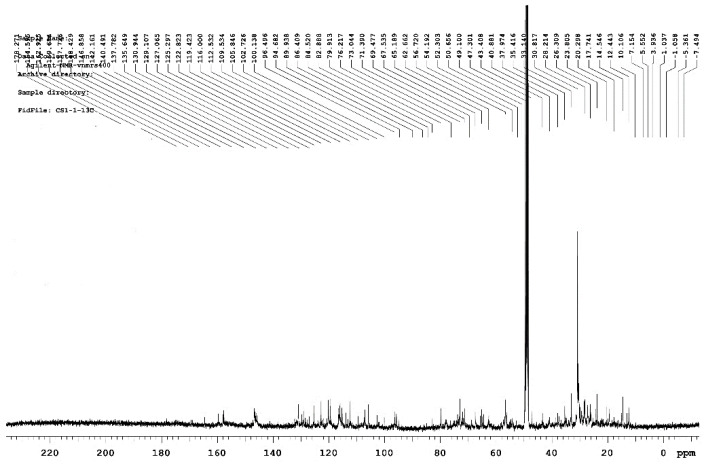
^13^C-NMR spectra of RPB sample.

Carbon is the skeletal element of organic compounds. It is very important to understand the structure of organic compounds by analyzing their carbon atoms. Some functional groups in organic compounds do not contain hydrogen. Thus, data of these functional groups cannot be obtained from the ^1^H spectrum, but only from the ^13^C spectrum. The ^1^H NMR chemical shift usually occurs from 0 to 15 ppm, whereas the usual range of the ^13^C NMR shift is from 0 to 300 ppm, which is about 20 times that of ^1^H. Therefore, the importance of ^13^C NMR analysis for chemical research has been clearly recognized [49,50]. Among them, the C of the saturated alkane is a sp^3^ hybrid that generally appeared at the 2.5–55 ppm position as shown in Figure 7. The first smaller peak in the figure appears around 14 ppm, which may be the delta values of the C atoms in butane and pentane. A larger peak appeared at the 31 ppm position, which was the delta value of C in neopentane and was more abundant. The largest peak at the 49 ppm position was determined to be the solvent peak in the CHCDO solvent at ^13^C. The C in the alkyne is a sp hybrid, and the main chemical shift occurred at 67–92 ppm. The smaller peaks appearing at 73 and 80 ppm in the figure may be the delta values for C atoms in the alkane species. The C of the olefin is sp^2^ hybridized with a major chemical shift value of 100–165 ppm. A large number of small peaks appeared in the 100–120 ppm range, which may have been caused by an olefinic substance C atom. The chemical shift in aromatic compound C atoms generally occurs at 120–160 ppm [51,52,53]. The peak appearing near 120–130 ppm represented the C atoms in an aromatic compound. In addition, C in a carbonyl group is closer to the lower field and generally occurred at the 160–220 ppm position.

**Figure 8:**
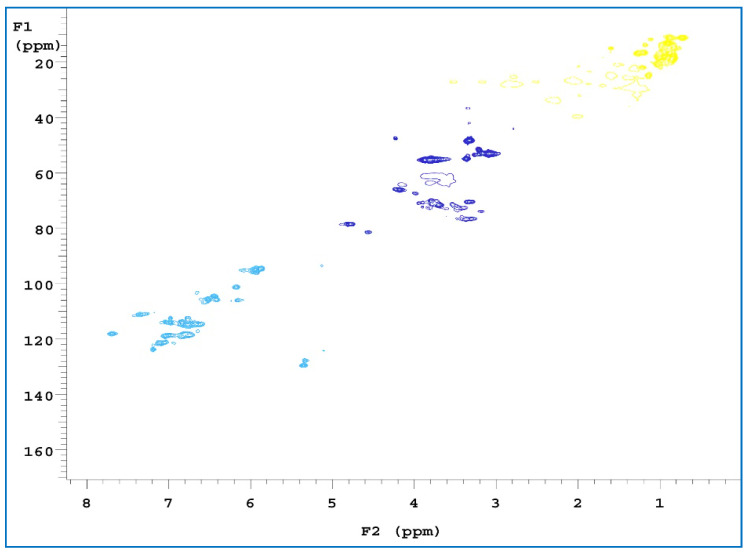
2D-HSQC NMR spectra of RPB methanol extract.

**Figure 9:**
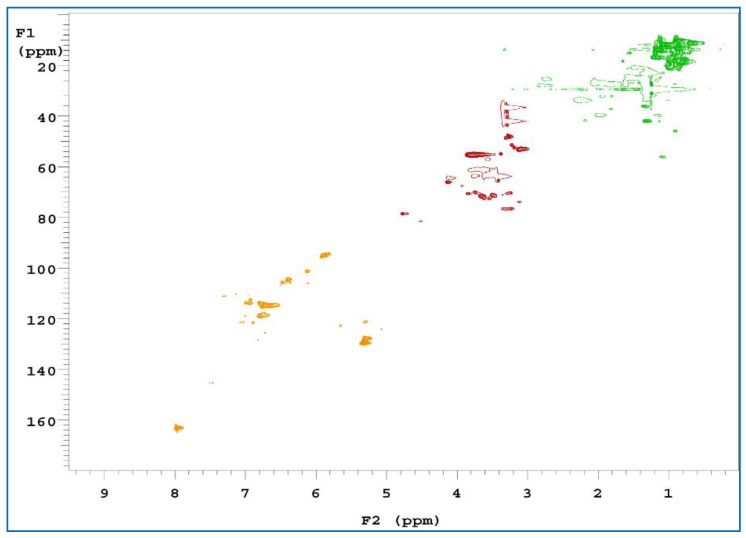
2D-HSQC NMR spectra of RPB ethanol/benzene extract.

**Figure 10:**
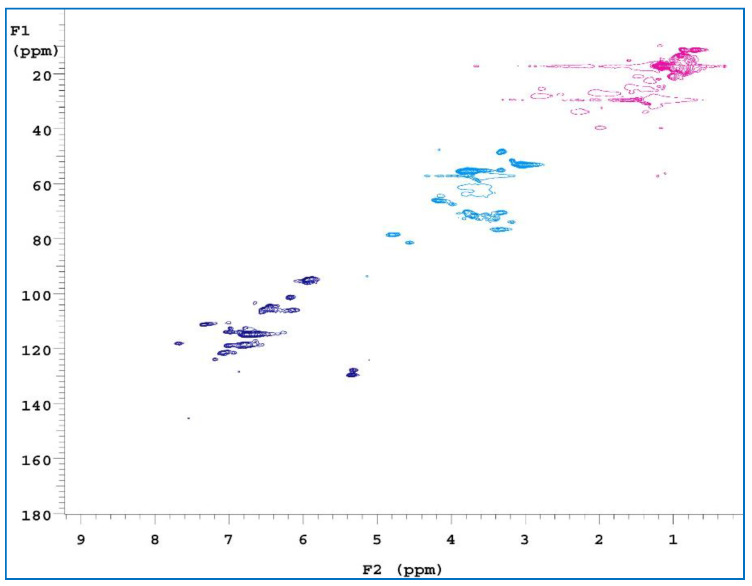
2D-HSQC NMR spectra of RPB ethanol/methanol extract.

2D-HSQC is one of the most common 2D NMR spectra, which provides information about both ^1^H and ^13^C cores and is important for structural analyses. It can also be used to detect the association between overlapping signals in ^1^H and ^13^C-NMR spectra [54,55]. To obtain further structural characterization information for the samples, three of the extracts were subjected to a 2D-HSQC NMR analysis. Comparing the three spectra (Figure 8, Figure 9 and Figure 10), C and H interacted with each other at three locations. The first location was the δ_H_ value, which was equal to 0.6–1.5 ppm, and the δ_C_ value was 10–30 ppm, where a signal point appeared. These locations revealed signals for some alkynes and some saturated hydrocarbons, such as olefins. The second location has a δ_H_ value of 3–4 ppm and a δ_C_ value of 40–80 ppm. The signals at these locations are for alcohols, ethers, phenols, and other oxygenated compounds [56]. The third location has a δ_H_ value of 6–7.5 ppm and a δ_C_ value of 90–130 ppm. These locations are signal points for aromatic compounds.

#### 3.2.3. GC-MS Analysis

**Figure 11:**
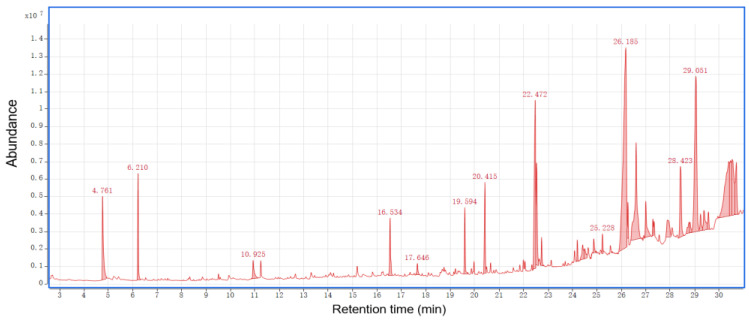
Total ion chromatograms of RPB methanol extract.

**Figure 12:**
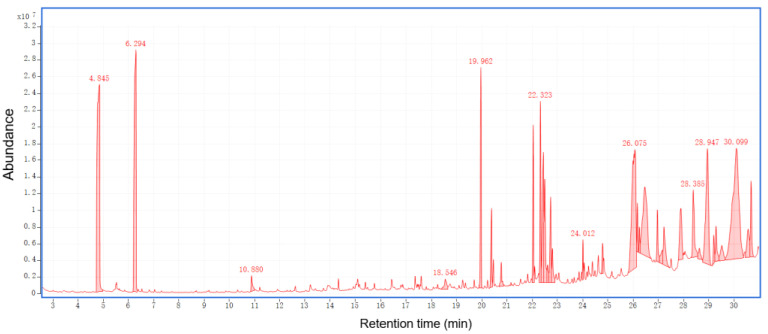
Total ion chromatograms of RPB ethanol/benzene (1:1) extract.

**Figure 13:**
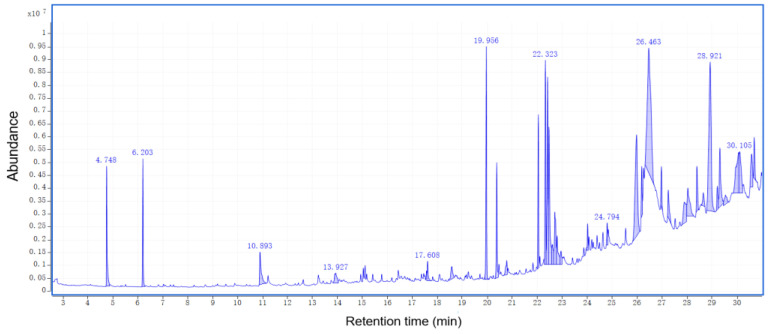
Total ion chromatograms of RPB ethanol/methanol extract.

The total ion chromatograms of RPB extractives analyzed by GC-MS were shown in Figure 11, Figure 12 and Figure 13. The peak area normalization method was used to calculate the content of each component, and specific results are shown in Tables S5–S7.

According to the GC-MS results, 32 peaks were detected in the bark methanol extract. The results show the percentages of the following substances: 17-pentatriacontene (21.58%), gamma-sitosterol (7.40%), (Z,Z)-9,12-octadecadienoic acid (7.24%), oleic acid (4.16%), formamide (3.13%), n-hexadecanoic acid (2.56%), 2-ethyl-1-hexanol (2.52%), and 2,6-bis(3,4-methylenedioxyphenyl)-3,7-dioxabicyclo (3.3.0) octane (11.90%).

According to the GC-MS results, 33 peaks were detected in the bark ethanol/benzene extract. The results show the percentages of the following substances: N,N-diethyl-3-formamide (11.55%), 2-ethyl-1-hexanol (9.98%), 2-methyl-hexadecanoic acid (3.78%), i-propyl 14-methyl-pentadecanoate (1.60%), heptadecanoic acid, 15-methyl-, methyl ester (3.12%), (Z)-18-octadec-9-enolide (3.99%), 1-heptatriacotanol (2.71%), ethyl stearate, mono 9-epoxy (0.64%), 1,22-docosanediol (10.26%), beta-sitosterol (7.10%), 17-pentatriacontene (2.43%), and (+)-sesamin (6.78%).

According to the GC-MS results, 36 peaks were detected in the bark ethanol/methanol extract. The results show the percentages of the following substances: N,N-diethyl-formamide (4.75%), 2-ethyl-1-hexanol (2.47%), indole (1.67%), hexadecanoic acid, methyl ester (5.10%), n-hexadecanoic acid (2.83%), methyl stearate (4.71%), (Z,Z)-9,12-octadecadienoic acid (5.60%), 1-heptatriacotanol (5.22%), gamma-sitosterol (14.40%), and 3-acetoxy-7,8-epoxylanostan-11-ol (1.22%).

A variety of organic materials are shown in Tables S5–S7. For example, the alcohols were 2-ethyl-1-hexanol, 1,22-docosanediol, and beta-sitosterol. The acid substances were n-hexadecanoic acid, oleic acid, (Z,Z)-9,12-octadecadienoic acid, and 9-octadecenoic acid. In addition, aromatic organic compounds were present, including indole, d-mannose, methyl stearate, and formamide. Among them, β-sitosterol lowers cholesterol, relieves cough, inhibits tumors, and repairs tissues, and is often used as a hypolipidemic drug [57,58,59]. The aromatic compound indole is often found in natural flower oil and is widely used in floral fragrances, such as jasmine, neroli, gardenia, lotus, narcissus, and white orchid. Indole is a raw material for spices, medicines, and plant growth hormone drugs [60,61]. Formamide is reactive and has specific solubility. It is also a raw material for synthetic medicines, spices, and dyes. It is a moderately toxic substance.

#### 3.2.4. Analysis of LC-QTOF-MS

In order to determine the composition of the compound of the bark extract more accurately, we performed a LC-QTOF-MS test analysis on the bark ethanol/methanol extract. LC-QTOF-MS is a rapid separation method with high accuracy and a wide separation range. It does little structural damage to compounds and is suitable for the separation of organic molecules and biomolecules [62].

According to the LC-QTOF-MS results, 256 peaks were detected in the bark ethanol/methanol extract. The results show the percentages of the following substances: Nb-Methyltetrahydroharman (24.68%), Bufotenidine (16.92%), 2-Methyl-6-methoxy-Erycibelline (5.62%), Patriscabratine (4.56%), Hypaphorine (4.02%), Betaine (3.04%), Echinopsine (2.29%), Corynantheine (2.25%), Indole (0.70%), Holadysine (0.44%), Pegamine (1.10%), 4-Methoxyl-1-methyl-2-quinolone (0.85%), Phthalic anhydride (0.42%), Patriscabratine (1.23%), L-Epigallocatechin (0.43%), Epicatechin (1.03%), and Didrovaltratum (0.99%).

As shown in Table S8, different organic compounds were detected. Among the many tested compounds, Betaine is an important substance detected. Betaine is a natural compound belonging to the quaternary ammonium alkaloid, which is named for its initial extraction from sugar beets. It is soluble in water, methanol and ethanol and is often found in plants such as beetroot and cottonseed. The addition of Betaine to the feed has the effect of protecting the vitamins in the feed. The feed can withstand high temperatures and long storage periods, which can greatly improve the utilization of the feed and can also reduce costs. It is clinically shared with N-mercapto glycine to treat myasthenia gravis. At the same time, studies have shown that Betaine also lowers blood fat, resists liver fat, and has an anti-aging effect [63,64]. Second, another compound, Phthalic anhydride, was also detected. Phthalic anhydride is an important organic chemical material that is industrially resistant to scorching and improves operational safety. At the same time, it is also a widely used acid anhydride resin curing agent, which is low in price and low in heat generation during curing and is particularly suitable for large-scale casting products and laminates. On the other hand, Epicatchin is also detected in the bark. Studies have shown that Epicatechin has the effects of clearing away heat and detoxifying purulent sputum and is clinically used for the treatment of pulmonary phlegm and pus, lung heat and cough, breast moth swelling and so on. Epicatechin also has a preventive effect on cardiovascular and cerebrovascular diseases and can prevent tumors and has an antioxidant effect. Epicatechin inhibits LPS-induced RAW264.7 cell inflammatory response [65,66] and plays an important role in the medical industry.

## 4. Conclusions

Adding the nanocatalyst resulted in a significant increase in the DTG curve during the experiment. The nanocatalyst accelerated the bark powder pyrolysis reaction to produce the bark biomass. More energy is created from the conversion of cellulose and hemicellulose to ethanol. The 3D-FT-IR test chart also showed such a feature. The energy released by cleavage of the C-H and C=O double bonds during pyrolysis is considered biomass energy. In the PY-GC-MS analysis, the small molecules of the bark components were mainly present in the 20–30 min pyrolysis stage, accounting for 58% of the molecular weight of the entire pyrolysis process. At the same time, the types and substance names of the pyrolysis molecules were obtained. Phenolics accounted for 32%. Studies have shown that the antioxidant phenolic compounds reduce lipid oxidation and protect against heart disease. In addition, phenolics have higher antioxidant capacity than vitamin E, which can effectively reduce infarctions caused by arterial stenosis. Hydrocarbons, ketones, sugars, alcohols and aldehydes were also detected.

FT-IR revealed that the absorption peaks of the RPB extracts were mainly concentrated in the 3730–3000 cm^−1^, 1750–1550 cm^−1^ and 1100–850 cm^−1^ bands. The main chemical components were phenols, alcohols, ethers, fatty acids, ketones, polysaccharides, and fatty acids. The cellulose absorption peak (3500–3300 cm^−1^) was significantly lower, indicating that a large amount of cellulose was hydrolyzed. The absorption peaks of hemicellulose (3400 and 2970 cm^−1^) and lignin (1470–1430 cm^−1^) were slightly lower, indicating that hemicellulose and lignin were less hydrolyzed. GC-MS detected 32, 33, and 36 peaks, in the three extracts, respectively. Among them, β-sitosterol lowers cholesterol, relieves cough, inhibits tumors, and repairs tissues. Aromatic compounds are often found in natural flower oils and are used as raw materials for perfumes, pharmaceuticals, and plant growth hormone drugs. The NMR H spectrum revealed that a chemical shift in protons mainly occurred at positions 1.3, 3.3, and 4.8 ppm. The chemical shift of protons mainly occurred near the 14, 49, and 100–120 ppm positions in the C spectrum. The 2D-HSQC spectrum showed the signal points at which C and H were related to each other.

RPB has great application prospects in the pharmaceutical and food industries. In addition, by adding nano-metal particles to the bark powder as a comparison, the nanocatalytic addition process of pyrolysis was added, and the pyrolysis efficiency of cellulose and hemicellulose was accelerated, which increased the efficiency of converting biomass oil into ethanol fuel. This study shows that *Robinia pseudoacacia* has a scientific basis for becoming a quality resource.

## Figures and Tables

**Table 1:** The functional group and classification of compounds obtained of RPB extracts.

Frequency Range (cm^−1^)	Frequency (cm^−1^)			Functional Group	Classification of Compounds
	Methanol Extract	Benzene/Ethanol Extract	Methanol/Ethanol Extract		
(1) 3500–3000	3434	3421	3413	O-H stretching	Alcohol, carboxylic acids
(2) 3000–2800	2915	2975	2982	C-H stretching	Alkane
(3) 1680–1610	1633	1653	1640	C=C stretching	Alkenes
(4) 1470–1340	-	1457, 1384	1457, 1384	C-H bending	Alkanes
(5) 1200–1000	1033	1087, 1047	1047	C-O stretching	Alcohol, ether, carboxylic acids
(6) 900–690		878	878	C-H out of plane bending	Aromatic rings

## Data Availability

The data presented in this study are available on request from the corresponding author.

## Supplementary Materials

The following are available online athttps://www.mdpi.com/article/10.3390/polym15010114/s1, Table S1: PY-GC-MS analysis of bark samples. Table S2: PY-GC-MS analysis of bark/Ag samples. Table S3: PY-GC-MS analysis of bark/Co_3_O_4_ samples. Table S4: PY-GC-MS analysis of bark/Ag+Co_3_O_4_ samples. Table S5: GC-MS analysis of bark methanol extract. Table S6: GC-MS analysis of bark ethanol/benzene extract. Table S7: GC-MS analysis of bark ethanol/methanol extract. Table S8 LC-QTOF-MS analysis of bark ethanol/methanol extract.
