# Supplementary material for: Nano Ag/Co3O4 Catalyzed Rapid Decomposition of Robinia pseudoacacia Bark for Production Biofuels and Biochemicals
Source: Polymers (Basel). 2022 Dec 27;15(1):114. doi: 10.3390/polym15010114 (PMC9824563; doi:10.3390/polym15010114)
Supplement: Supplementary file 1 [file polymers-15-00114-s001.zip › polymers-2007157-supplementary.pdf]

Supplementary Information

Table S1. PY-GC-MS analysis of bark samples

No.	Retention Time (min)	Peak Area (%)	Component
1	4.51	2.94	Carbon dioxide
2	4.73	0.04	2-Octanamine
3	5.51	1.83	Cyclopropylacetylene
4	5.86	0.08	Acetic acid
5	6.79	0.32	1,3-Cyclopentadiene, 1-methyl-
6	7.15	5.64	Benzene
7	8.92	0.05	1H-Pyrrole, 1-methyl-
8	9.07	0.22	Pyridine
9	9.22	0.23	2-Pentyn-4-one
10	9.62	4.74	Toluene
11	10.67	0.06	1,3,5-Cycloheptatriene
12	11.15	0.04	Pyridine, 4-methyl-
13	11.70	0.14	1H-Pyrrole, 3-methyl-
14	11.99	0.11	1H-Pyrrole, 2-methyl-
15	12.50	0.70	Ethylbenzene
16	12.75	0.64	p-Xylene
17	12.98	0.59	Phenylethyne
18	13.34	0.03	2,3,4,5-Tetrahydropyridazine
19	13.36	0.05	Cyclopentane, butyl-
20	13.46	1.97	Styrene
21	13.97	0.05	3-Dimethylaminoacrylonitrile
22	14.50	0.24	3H-Pyrazol-3-one, 1,2-dihydro-5-methyl-
23	14.78	0.03	(E)-1,1-Difluoro-2-ethylidenecycopropane
24	14.89	0.10	1H-Pyrrole, 2,5-dimethyl-
25	15.13	0.09	2,3-Pentadiene
26	15.46	0.05	2-Furancarbonitrile
27	15.57	0.05	Cyclopentanone, 3,4-bis(methylene)-
28	15.80	0.16	Benzene, (1-methylethyl)-
29	15.93	0.08	Cyclohexane, 1-ethenyl-2-methyl-, trans-
30	16.24	2.23	Phenol
31	16.47	0.28	2-indanol trifluoroacetate ester
32	16.59	0.25	Benzonitrile
33	16.64	0.16	Cyclopropane, nonyl-
34	16.80	0.04	2,3,4,5-Tetrahydropyridazine
35	16.89	0.36	Benzene, 1-ethenyl-3-methyl-
36	16.97	0.24	Benzene, 1,1'-(1-ethenyl-1,3-propanediyl)bis-
37	17.02	0.54	Benzofuran
38	17.64	0.04	Pyridine, 4-methyl-

39	17.91	0.16	1,2-Cyclopentanedione, 3-methyl-
40	18.02	0.05	1H-Imidazole, 1,2-dimethyl-
41	18.22	0.07	1,3-cis,5-cis-Octatriene
42	18.37	0.20	2-Ethylcyclohexanol,c&t
43	18.53	0.04	2-Hexene, 3-methyl-, (Z)-
44	18.62	3.46	Benzene, 1-propynyl-
45	18.93	0.24	Ethanone, 1-(1H-pyrrol-2-yl)-
46	19.04	0.07	Glutaric acid, tridec-2-yn-1-yl 2-ethylhexyl ester
47	19.10	0.04	3-Methyl-hexanoic acid
48	19.24	2.53	Phenol, 3-methyl-
49	19.39	0.04	1-But-1-enylaziridine
50	19.73	0.25	Ethylene diacrylate
51	19.83	0.83	Phenol, 2-methoxy-
52	19.97	0.16	1-Hexen-3-ol
53	20.08	0.34	4-Pyridinol
54	20.28	0.18	Benzofuran, 7-methyl-
55	20.42	0.15	Pyrido[2,3-d]pyrimidine
56	20.48	0.04	Maltol
57	20.61	0.33	2(1H)-Pyridinone, 3-methyl-
58	20.79	0.04	Propanoic acid, 2-(hydroxyimino)-, methyl ester
59	20.82	0.04	Cyclobuta[1,2:3,4]dicyclopentene, 1,3a,3b,4,6a,6b-hexahydro-
60	20.97	0.06	Phenol, 2-ethyl-
61	21.10	0.10	Indolizine
62	21.15	0.23	Benzyl nitrile
63	21.23	0.49	Phenol, 2,4-dimethyl-
64	21.35	0.20	Benzofuran, 2,3-dihydro-
65	21.49	0.77	Naphthalene, 1,2-dihydro-
66	21.57	0.09	Cyclohexanone, 4-(benzoyloxy)-, oxime
67	21.64	0.78	Phenol, 4-ethyl-
68	21.70	0.65	Phenol, 3-ethyl-
69	21.78	0.93	Methyl .beta.-d-ribofuranoside
70	21.89	0.11	2a,4a,6a,6b-Tetrahydrocyclopenta[cd]pentalene
71	21.96	0.09	Phenol, 2,3-dimethyl-
72	22.09	0.04	2-Methoxy-5-methylphenol
73	22.16	0.07	Benzenamine, 4-propoxy-
74	22.20	0.24	1-Undecanol
75	22.30	0.63	10-(Tetrahydro-pyran-2-yloxy)-tricyclo[4.2.1.1(2,5)]decan-9-ol
76	22.38	6.84	Catechol
77	22.60	0.46	Benzene, (ethenyloxy)-
78	22.79	2.14	Benzofuran, 2,3-dihydro-
79	22.97	0.15	m-Guaiacol
80	23.02	0.22	4-Fluorophenylhydrazine

81	23.16	0.53	Phenol, 2-propoxy-
82	23.25	0.30	Phenol, 3-ethyl-5-methyl-
83	23.34	0.10	2-Coumaranone
84	23.42	0.48	4H-1-Benzopyran-4-one, 2,3-dihydro-6-methyl-
85	23.54	0.47	Isoquinoline
86	23.61	0.19	D-Gluconic acid, .delta.-lactone
87	23.69	1.03	1,2-Benzenediol, 3-methyl-
88	23.84	1.47	1,2-Benzenediol, 3-methoxy-
89	23.99	0.09	Isoquinoline
90	24.22	2.48	1,2-Benzenediol, 4-methyl-
91	24.31	0.17	1H-Indol-3-amine
92	24.49	1.99	Indole
93	24.61	1.24	Naphthalene, 2-methyl-
94	24.69	0.06	2,3-Dimethylhydroquinone
95	24.79	2.82	2-Methoxy-4-vinylphenol
96	24.88	0.11	6-Hydroxymethyl-5-methyl-bicyclo[3.1.0]hexan-2-one
97	24.94	0.82	Naphthalene, 1-methyl-
98	24.99	0.04	1-Naphthalenol, 5,8-dihydro-
99	25.07	0.24	2-Methylresorcinol, acetate
100	25.13	1.03	Phenol, 4-(2-propenyl)-
101	25.26	0.21	2-Cyclopropylthiophene
102	25.31	0.45	Benzenemethanol, .alpha.-ethynyl-
103	25.40	2.52	Phenol, 2,6-dimethoxy-
104	25.49	0.83	1H-Indenol
105	25.61	0.70	Phenol, 3,4-dimethoxy-
106	25.69	0.18	Phenol, 2-methoxy-4-propyl-
107	25.77	0.44	Ethanethioic acid, S-phenyl ester
108	25.84	0.43	1,3-Benzenediamine, 4-methoxy-
109	26.01	0.37	1,4-Benzenediol, 2,6-dimethyl-
110	26.09	0.32	1H-Indole, 2-methyl-
111	26.25	1.63	4-Hydroxy-2-methoxybenaldehyde
112	26.35	0.26	trans-Isoeugenol
113	26.47	0.51	Pyrrole-3-carbonitrile, 5-formyl-2,4-dimethyl-
114	26.72	0.18	Naphthalene, 1,7-dimethyl-
115	26.91	1.54	3,5-Dimethoxy-4-hydroxytoluene
116	27.01	3.48	trans-Isoeugenol
117	27.17	0.33	1,2-Dimethylbenzimidazole
118	27.30	1.02	Biphenylene
119	27.57	1.54	.beta.-D-Glucopyranose, 1,6-anhydro-
120	27.77	0.04	Benzaldehyde, 2,5-dimethoxy-
121	27.88	0.06	Phenol, 4-(aminomethyl)-2-methoxy-
122	28.03	0.11	3-Pyridinemethanol, 5-hydroxy-4,6-dimethyl-
123	28.08	0.61	1-Naphthalenol

124	28.22	1.58	1,1'-Biphenyl, 2-ethyl-
125	28.31	0.27	Benzenamine, 4-methoxy-2-methyl-
126	28.39	1.24	2-Propanone, 1-(4-hydroxy-3-methoxyphenyl)-
127	28.53	0.81	1-Oxa-4-thiaspiro[4.5]decane
128	28.75	0.20	Dihydro iso-jasmone
129	28.85	0.05	2-Methyl-1-phenyl-1-butanol
130	28.91	2.38	2,3,5,6-Tetrafluoroanisole
131	29.32	0.26	Ethanone, 1-(2-hydroxy-6-methoxyphenyl)-
132	29.58	0.86	Phenol, 2,6-dimethoxy-4-(2-propenyl)-
133	29.68	0.60	Fluorene
134	30.13	0.15	1,4-epoxynaphthalene, 1,4-dihydro-1-methyl-
135	30.38	0.27	Bi-1,3,5-cycloheptatrien-1-yl
136	30.58	1.28	2,5-Dimethoxy-4-ethylbenzaldehyde
137	30.70	0.41	pentan-2-yl phenyl ether
138	30.84	0.11	Benzaldehyde, 4-hydroxy-3,5-dimethoxy-
139	31.69	2.05	(E)-2,6-Dimethoxy-4-(prop-1-en-1-yl)phenol
140	32.07	0.03	Tellurophene
141	32.11	0.05	4-Chloro-3-methylbenzoic acid
142	32.37	1.30	4,5-Dihydro-1-phenyl-1H-furo(2,3-c)pyrazole
143	32.58	0.47	Ethanone, 1-(4-hydroxy-3,5-dimethoxyphenyl)-
144	32.71	0.61	4-((1E)-3-Hydroxy-1-propenyl)-2-methoxyphenol
145	33.28	0.05	Propenoic acid, 3-(bicyclo[2.2.1]hept-1-yl)-, methyl ester
146	33.36	0.09	1-Hexacosanol
147	33.43	0.15	2-Benzylidenecyclopentanone
148	33.54	0.17	2-Pentanone, 1-(2,4,6-trihydroxyphenyl)
149	33.73	0.33	1-Tetradecanol
150	34.62	0.32	Anthracene
151	34.92	0.11	9H-Fluorene, 9-methylene-
152	35.22	0.04	Cinnamic acid, .beta.-chloro-, (Z)-
153	35.83	0.09	Bicyclo[2.2.2]octane, 1-iodo-4-phenyl-
154	36.22	0.05	5H-Indeno[1,2-b]pyridine
155	39.82	0.10	Thiophene-3-carboxaldehyde, 5-chloro-2-dihydroxyboryl-
156	40.12	0.20	1,9-Diazaspiro(4,4)nonane-2,8-dione

Table S2. PY-GC-MS analysis of bark/Ag samples

No.	Retention Time (min)	Peak Area (%)	Component
1	5.74	16.74	2-Butene
2	5.88	3.09	Acetaldehyde
3	5.99	1.85	Cyclopropane, ethyl-

4	6.21	2.75	Furan
5	6.46	9.93	Acetone
6	6.53	3.61	1-Pentene, 4-methyl-
7	6.65	0.12	Butane, 2,3-dimethyl-2-nitro-
8	6.8	3.14	Acetonitrile
9	6.9	3.27	Propanal, 2-methyl-
10	6.99	2.51	Furan, 2-methyl-
11	7.43	6.44	Acetic acid ethenyl ester
12	7.61	2.11	1-Heptene
13	7.72	0.69	Cyclohexene
14	7.9	3.04	Benzene
15	8.27	1.95	Butanal, 3-methyl-
16	8.36	2.20	Butanal, 2-methyl-
17	8.52	4.66	Acetic acid
18	8.81	0.10	8,11,14-Eicosatrienoic acid, methyl ester, (Z,Z,Z)-
19	8.94	0.13	Tetraacetyl-d-xylonic nitrile
20	9.03	0.44	Pentanal
21	9.13	0.16	Tetraacetyl-d-xylonic nitrile
22	9.33	0.90	2-Octene
23	9.53	0.11	-
24	9.82	4.40	Toluene
25	10.02	1.32	1H-Pyrrole, 1-methyl-
26	10.2	0.72	Pyridine
27	10.36	0.66	Borane, diethylmethyl-
28	10.49	0.18	Pyrazole[4,5-b]imidazole, 1-formyl-3-ethyl-6-?d-ribofuranosyl-
29	10.67	0.62	3-Pentanol, 3-methyl-
30	10.89	0.12	Phorbol
31	11.16	0.41	Olean-12-ene-3,15,16,21,22,28-hexol
32	11.41	0.68	3-Undecene, (Z)-
33	11.67	2.58	Pyrrole
34	11.96	0.30	PyridinePhenylalanine, 4-amino-N-t-butyloxycarbonyl-, t-butyl ester
35	12.28	0.24	Olean-12-ene-3,15,16,21,22,28-hexol
36	12.38	0.15	-
37	12.5	0.72	Acetic anhydride
38	12.7	0.32	1,3,5,7-Cyclooctatetraene
39	12.9	1.13	Furfural
40	13.32	0.32	Olean-12-ene-3,15,16,21,22,28-hexol
41	13.64	0.53	2,3-Butanedione
42	13.83	0.16	-
43	13.96	0.07	Olean-12-ene-3,15,16,21,22,28-hexol
44	14.06	0.27	Olean-12-ene-3,15,16,21,22,28-hexol

45	14.31	0.26	Olean-12-ene-3,15,16,21,22,28-hexol
46	14.48	0.08	-
47	14.54	0.15	9-Octadecen-12-ynoic acid, methyl ester
48	14.67	0.42	E-2-Hexenyl benzoate
49	14.81	0.23	9-Octadecen-12-ynoic acid, methyl ester
50	15.07	0.28	9-Octadecen-12-ynoic acid, methyl ester
51	15.15	0.13	Phorbol
52	15.19	0.21	2(5H)-Furanone
53	15.32	0.15	d-Mannitol, 1-decylsulfonyl-
54	15.4	0.03	d-Mannitol, 1-decylsulfonyl-
55	15.58	0.47	Octadecanal, 2-bromo-
56	15.71	0.52	Phenol
57	15.79	0.14	cis-1,2-Dihydrocatechol
58	15.93	0.91	Phenol, 2-methoxy-
59	16.25	0.54	Phorbol
60	16.43	0.28	-
61	16.63	0.51	1,3-Benzenediol, 4-ethyl-
62	16.8	0.23	Phorbol
63	16.92	0.02	Olean-12-ene-3,15,16,21,22,28-hexol
64	17.04	0.05	9-Octadecen-12-ynoic acid, methyl ester
65	17.07	0.04	1,4-Benzenediol, 2,6-bis(1,1-dimethylethyl)-
66	17.12	0.06	1,4-Benzenediol, 2,6-bis(1,1-dimethylethyl)-
67	17.2	0.03	9-Octadecen-12-ynoic acid, methyl ester
68	17.35	0.28	2-Nonenal, (E)-
69	17.48	0.81	N-ethyl-, ethyl-3,4-methylenedioxyphenethylamine
70	17.62	0.09	Phorbol
71	17.69	0.10	Pregn-4-ene-3,20-dione, 11-hydroxy-
72	17.84	0.69	5H-1-Pyrindine
73	17.99	0.06	1,4-Benzenediol, 2,6-bis(1,1-dimethylethyl)-
74	18.17	0.04	5,3-Acetoxypregnan-20-ol-6-one-18-oic acid lactone
75	18.28	0.23	1,3-Dimethyl-3-hydroxy-5-methoxyoxindole
76	18.33	0.07	Benzene, methanol, ethynyl-,phenyl-
77	18.44	0.18	Ethyl iso-allocholate
78	18.51	0.02	2,4,6-Cycloheptatrien-1-one, 3,5-bis-trimethylsilyl-
79	18.71	0.04	Betamethasone acetate
80	18.81	0.04	Cholan-24-oic acid, 3,7-dioxo-
81	18.86	0.03	1,2-Benzisothiazol-3-amine
82	18.94	0.03	Olean-12-ene-3,15,16,21,22,28-hexol
83	19.06	0.22	-
84	19.14	0.08	6,Hydroxyfluoxymesterone
85	19.25	0.07	1-Isopropoxy-5-propyl-2,3-bis-trimethylsilyl-1,2-

			dihydroborinine
86	19.3	0.04	1,4-Benzenediol, 2,6-bis(1,1-dimethylethyl)-
87	19.33	0.06	1-Decene
88	19.43	0.07	Olean-12-ene-3,15,16,21,22,28-hexol
89	19.55	0.11	Olean-12-ene-3,15,16,21,22,28-hexol
90	19.62	0.02	1,4-Benzenediol, 2,6-bis(1,1-dimethylethyl)-
91	19.67	0.03	Olean-12-ene-3,15,16,21,22,28-hexol
92	19.73	0.05	-
93	19.77	0.07	Olean-12-ene-3,15,16,21,22,28-hexol
94	19.86	0.02	9-Desoxo-9-x-acetoxy-3,8,12-tri-O-acetylingol
95	19.9	0.02	Olean-12-ene-3,15,16,21,22,28-hexol
96	19.96	0.03	Olean-12-ene-3,15,16,21,22,28-hexol
97	20.04	0.03	Olean-12-ene-3,15,16,21,22,28-hexol
98	20.22	0.04	11-Deoxycortisol, bis(O-ethyloxime)
99	20.73	0.11	3Beta,17beta-diacetoxy-5-chloro-6beta-nitro-5alpha-androstane
100	23.06	0.12	5,7,9(11)-Androstatriene, 3-hydroxy-17-oxo-
101	25.51	0.13	Ursodeoxycholic acid
102	25.84	0.10	Betulin
103	25.98	0.10	Phenol, 2-methoxy-4-propyl-
104	26.05	0.07	Oleanolic acid

Table S3. PY-GC-MS analysis of bark/Co_3O_4 samples

No.	Retention Time (min)	Peak Area (%)	Component
1	5.74	19.22	2-Butene
2	5.89	3.74	Acetaldehyde
3	5.99	1.17	Cyclopropane, 1,1-dimethyl-
4	6.21	2.64	Furan
5	6.46	14.59	Acetone
6	6.66	0.07	Cyclopropanecarboxamide
7	6.8	3.05	Methyl isocyanide
8	6.91	3.85	Propanal, 2-methyl-
9	6.99	2.59	Furan, 2-methyl-
10	7.25	0.73	4-Methyl-2-pentyne
11	7.43	6.12	Guanidine
12	7.61	2.31	1-Heptene
13	7.72	0.68	(Z),(Z)-2,4-Hexadiene
14	7.9	2.96	Benzene
15	8.1	0.01	Norpseudoephedrine
16	8.26	2.12	Butanal, 3-methyl-
17	8.35	2.40	Butanal, 2-methyl-
18	8.53	1.80	Acetic acid

19	8.64	1.13	Methacrolein
20	8.81	0.08	3-Octyn-2-ol
21	8.93	0.14	Cyclopropyl carbinol
22	9.03	0.37	Cyclopropyl carbinol
23	9.13	0.15	Cyclopropyl carbinol
24	9.32	0.92	1-Octene, 3,7-dimethyl-
25	9.52	0.19	Azetidine, 1-nitroso-
26	9.67	0.01	Cyclopropyl carbinol
27	9.82	4.22	Toluene
28	10.02	1.26	1H-Pyrrole, 1-methyl-
29	10.19	0.61	Pyridine
30	10.35	0.59	Octanedinitrile
31	10.48	0.15	-
32	10.67	0.57	Butanenitrile, 3-methyl-
33	10.87	0.16	Phorbol
34	11.15	0.37	Olean-12-ene-3,15,16,21,22,28-hexol
35	11.42	0.46	1-Nonene
36	11.5	0.13	Olean-12-ene-3,15,16,21,22,28-hexol
37	11.67	2.52	Pyrrole
38	11.95	0.29	o-Xylene
39	12.25	0.16	Olean-12-ene-3,15,16,21,22,28-hexol
40	12.36	0.18	1-(4-Chlorophenyl)-5-[(2-piperidin-1-yl)vinyl]-1H-tetrazole
41	12.5	0.40	Cyclobutylamine
42	12.69	0.39	1,3,5,7-Cyclooctatetraene
43	12.91	1.00	Furfural
44	13.33	0.27	2-Decene, (Z)-
45	13.62	0.27	Cyclobutylamine
46	14.05	0.13	Olean-12-ene-3,15,16,21,22,28-hexol
47	14.3	0.11	Olean-12-ene-3,15,16,21,22,28-hexol
48	14.43	0.02	Olean-12-ene-3,15,16,21,22,28-hexol
49	14.69	0.32	6-(Hydroxy-phenyl-methyl)-2,2-dimethyl-cyclohexanone
50	14.83	0.21	1,4-Benzenediol, 2,6-bis(1,1-dimethylethyl)-
51	15.58	0.32	Octadecane, 1-(ethenyloxy)-
52	15.72	0.39	Phenol
53	15.93	0.56	Phenol, 2-methoxy-
54	16.26	0.29	9-Octadecen-12-ynoic acid, methyl ester
55	16.3	0.30	Phenol, 3-methyl-
56	16.45	0.24	N-Cbz-glycyl-L-tyrosine benzyl ester
57	16.62	0.36	2-Methoxy-6-methylphenol
58	16.81	0.17	9-Octadecen-12-ynoic acid, methyl ester
59	17.33	0.35	Silane, trichlorodocosyl-
60	17.47	0.86	2-Methoxy-4-vinylphenol

61	17.7	0.32	Phenol, 2,6-dimethoxy-
62	17.85	0.79	5H-1-Pyrindine
63	18.25	0.25	Phenol, 2-methoxy-4-(1-propenyl)-
64	18.29	0.24	Phenol, 2-methoxy-4-(1-propenyl)-
65	18.38	0.12	9-Octadecen-12-ynoic acid, methyl ester
66	18.42	0.15	7-Heptadecene, 17-chloro-
67	19.06	0.30	Hexadecane, 1,1-bis(dodecyloxy)-
68	20.66	0.21	Hexadecane, 1,1-bis(dodecyloxy)-
69	20.79	0.49	[1,4]dioxino[2,3-b:5,6-b']dipyridine
70	21.73	0.16	Octadecanal, 2-bromo-
71	23.06	0.24	17-Pentatriacontene
72	23.83	0.15	Boroxin, tris(2,3-dimethylbut-2-yl)-
73	24.72	0.10	Vitexin
74	26.62	0.07	Chromone, 5-hydroxy-6,7,8-trimethoxy-2,3-dimethyl-
75	26.71	0.02	1,4-Benzenediol, 2,6-bis(1,1-dimethylethyl)-
76	26.83	0.18	Cyclohexane, 1,3,5-trimethyl-2-octadecyl-

Table S4. PY-GC-MS analysis of bark/Ag+Co_3O_4 samples

No.	Retention Time (min)	Peak Area (%)	Component
1	5.75	14.46	2-Butene
2	5.9	2.93	Acetaldehyde
3	6.01	0.97	Cyclopropane, 1,1-dimethyl-
4	6.22	2.62	Furan
5	6.48	15.76	Acetone
6	6.67	0.06	Butane, 2,3-dimethyl-2-nitro-
7	6.81	4.09	Methyl isocyanide
8	6.92	3.13	Propanal, 2-methyl-
9	7.0	2.26	Furan, 2-methyl-
10	7.25	0.43	3-Methyl-4-propenyl-oxetan-2-one
11	7.44	6.85	Acetic acid ethenyl ester
12	7.62	2.48	1-Heptene
13	7.72	0.70	Cyclohexene
14	7.91	3.37	Benzene
15	8.27	2.37	Butanal, 3-methyl-
16	8.36	2.39	Butanal, 2-methyl-
17	8.52	2.20	Acetic acid
18	8.62	1.35	2,4-Hexadienal, (E,E)-
19	8.82	0.09	3-Octyn-2-ol
20	9.03	0.75	Pentanal
21	9.13	0.20	2,3-Pentanedione
22	9.33	1.14	2-Octene

23	9.52	0.25	Azetidine, 1-nitroso-
24	9.82	4.50	Toluene
25	10.02	1.52	1H-Pyrrole, 1-methyl-
26	10.2	0.89	Pyridine
27	10.36	0.74	Heptanedinitrile
28	10.49	0.18	Pyrazole[4,5-b]imidazole, 1-formyl-3-ethyl-6-d-ribofuranosyl-
29	10.67	0.70	Propane, 2-nitro-
30	10.89	0.22	Phorbol
31	11.05	0.12	-
32	11.15	0.51	9-Octadecen-12-ynoic acid, methyl ester
33	11.41	0.69	1-Nonene
34	11.67	2.97	Pyrrole
35	11.92	0.31	Phenylalanine, 4-amino-N-t-butyloxycarbonyl-, t-butyl ester
36	12.35	0.08	3-Furaldehyde
37	12.4	0.05	Olean-12-ene-3,15,16,21,22,28-hexol
38	12.5	0.47	Cyclobutylamine
39	12.68	0.41	1,3,5,7-Cyclooctatetraene
40	12.9	1.24	Furfural
41	13.14	0.03	Olean-12-ene-3,15,16,21,22,28-hexol
42	13.33	0.29	1-Decene
43	13.63	0.35	1,2-Ethanediol, diacetate
44	13.99	0.10	Olean-12-ene-3,15,16,21,22,28-hexol
45	14.66	0.39	4-Benzoyloxy-1-morpholinocyclohexene
46	14.81	0.29	Olean-12-ene-3,15,16,21,22,28-hexol
47	15.17	0.43	Olean-12-ene-3,15,16,21,22,28-hexol
48	15.55	0.36	Octadecane, 3-ethyl-5-(2-ethylbutyl)-
49	15.71	0.33	Carbamic acid, N-(3-ethylphenyl)-, phenyl ester
50	15.94	0.55	Phenol, 2-methoxy-
51	16.23	0.24	Agaricic acid
52	16.3	0.10	Phenol, 3-methyl-
53	16.37	0.18	Olean-12-ene-3,15,16,21,22,28-hexol
54	16.62	0.48	Creosol
55	16.82	0.16	Phorbol
56	17.33	0.33	Undecanal, 2-methyl-
57	17.46	0.79	2-Methoxy-4-vinylphenol
58	17.71	0.52	Phenol, 2,6-dimethoxy-
59	17.83	0.66	m-Aminophenylacetylene
60	18.28	0.33	Phenol, 2-methoxy-4-(1-propenyl)-
61	18.39	0.19	Cyclopropanedodecanoic acid, 2-octyl-, methyl ester
62	19.05	0.17	Tetrabutyl titanate
63	19.1	0.12	9-Desoxo-9-x-acetoxy-3,8,12-tri-O-acetylingol

64	20.31	0.02	Oleic acid, eicosyl ester
65	20.76	0.43	[1,4]dioxino[2,3-b:5,6-b']dipyridine
66	20.93	0.07	Olean-12-ene-3,15,16,21,22,28-hexol
67	21.73	0.16	Hexadecanoic acid, 2-bromo-
68	21.85	0.03	-
69	21.89	0.03	Olean-12-ene-3,15,16,21,22,28-hexol,
70	22.06	0.06	5'-S-Methyl-5'-thioadenosine, N,O,O'-tris(trifluoroacetyl)-
71	22.95	0.05	Olean-12-ene-3,15,16,21,22,28-hexol
72	23.06	0.09	Agaricic acid
73	23.12	0.02	-
74	23.17	0.02	Ursodeoxycholic acid
75	23.54	0.03	N,N'-Ethylenebis(2-[2-hydroxyphenyl]glycine)
76	23.72	0.04	1-Isopropoxy-5-propyl-2,3-bis-trimethylsilyl-1,2-dihydroborinine
77	24.14	0.02	Cholestane, 2,3-epoxy-,
78	24.54	0.04	Digoxigenin
79	24.59	0.07	-
80	24.72	0.03	Chromone, 5-hydroxy-6,7,8-trimethoxy-2,3-dimethyl-
81	25.6	0.03	Olean-12-ene-3,15,16,21,22,28-hexo
82	25.76	0.05	9,12,15-Octadecatrienoic acid, 2-phenyl-1,3-dioxan-5-yl ester
83	26.2	0.03	6-Hydroxyfluoxymesterone
84	26.46	0.02	Olean-12-ene-3,15,16,21,22,28-hexol
85	26.99	0.02	Olean-12-ene-3,15,16,21,22,28-hexol

Table S5. GC-MS analysis of bark methanol extract**.**

No.	Retention Time (min)	Peak Area (%)	Component
1	4.761	3.13	Formamide
2	6.21	2.52	1-Hexanol, 2-ethyl-
3	10.925	0.90	Indole
4	16.534	1.81	decahydro-.alpha.,.alpha.,4a-trimethyl-8-methylene-, [2R-(2.alpha.,4a.alpha.,8a.beta.)]-2-Naphthalenemethanol
5	17.646	0.44	(E)-4-(3-Hydroxyprop-1-en-1-yl)-2-methoxyphenol
6	19.594	1.70	6-Isopropenyl-4,8a-dimethyl-1,2,3,5,6,7,8,8a-octahydro-naphthalen-2-ol
7	20.415	2.56	n-Hexadecanoic acid
8	22.472	7.24	9,12-Octadecadienoic acid (Z,Z)-
9	22.524	4.16	Oleic Acid
10	22.737	0.74	Dasycarpidan-1-methanol, acetate (ester)
11	24.199	0.47	4-[7-Acetoxy-2,2-dimethyl-3a-(2-oxo-ethyl)-

			tetrahydro-[1,3]dioxolo[4,5-c]pyran-6-yl]-3-methyl-but-2-enoic acid, methyl, ester
12	24.419	0.88	4H-Cyclopropa[5',6']benz[1',2':7,8]azuleno[5,6-b]oxiren-4-one, 8-(acetyloxy)-1,1a,1b,1c,2a,3,3a,6a,6b,7,8,8a-dodecahydro-3a,6b,8a-trihydroxy-2a-(hydroxymethyl)-1,1,5,7-tetramethyl-, (1a.alpha.,1b.beta.,1c.beta.,2a.beta.,3a.beta.,6a.alpha.,6b.alpha.,7.alpha.,8.beta.,8a.alpha.)-
13	24.503	0.58	4H-Cyclopropa[5',6']benz[1',2':7,8]azuleno[5,6-b]oxiren-4-one, 8-(acetyloxy)-1,1a,1b,1c,2a,3,3a,6a,6b,7,8,8a-dodecahydro-3a,6b,8a-trihydroxy-2a-(hydroxymethyl)-1,1,5,7-tetramethyl-, (1a.alpha.,1b.beta.,1c.beta.,2a.beta.,3a.beta.,6a.alpha.,6b.alpha.,7.alpha.,8.beta.,8a.alpha.)-
14	24.872	0.47	4H-Cyclopropa[5',6']benz[1',2':7,8]azuleno[5,6-b]oxiren-4-one, 8-(acetyloxy)-1,1a,1b,1c,2a,3,3a,6a,6b,7,8,8a-dodecahydro-3a,6b,8a-trihydroxy-2a-(hydroxymethyl)-1,1,5,7-tetramethyl-, (1a.alpha.,1b.beta.,1c.beta.,2a.beta.,3a.beta.,6a.alpha.,6b.alpha.,7.alpha.,8.beta.,8a.alpha.)-
15	25.228	0.49	4H-Cyclopropa[5',6']benz[1',2':7,8]azuleno[5,6-b]oxiren-4-one, 8-(acetyloxy)-1,1a,1b,1c,2a,3,3a,6a,6b,7,8,8a-dodecahydro-3a,6b,8a-trihydroxy-2a-(hydroxymethyl)-1,1,5,7-tetramethyl-, (1a.alpha.,1b.beta.,1c.beta.,2a.beta.,3a.beta.,6a.alpha.,6b.alpha.,7.alpha.,8.beta.,8a.alpha.)-
16	26.185	21.58	17-Pentatriacontene
17	26.269	0.86	4H-Cyclopropa[5',6']benz[1',2':7,8]azuleno[5,6-b]oxiren-4-one, 8-(acetyloxy)-1,1a,1b,1c,2a,3,3a,6a,6b,7,8,8a-dodecahydro-3a,6b,8a-trihydroxy-2a-(hydroxymethyl)-1,1,5,7-tetramethyl-, (1a.alpha.,1b.beta.,1c.beta.,2a.beta.,3a.beta.,6a.alpha.,6b.alpha.,7.alpha.,8.beta.,8a.alpha.)-
18	26.606	7.40	.gamma.-Sitosterol
19	27	1.73	4H-Cyclopropa[5',6']benz[1',2':7,8]azuleno[5,6]oxiren-4-one, 8,8a-bis(acetyloxy)-2a-[(acetyloxy)methyl]-1,1a,1b,1c,2a,3,3a,6a,6b,7,8,8a-dodecahydro-6b-hydroxy-3a-methoxy-1,1,5,7-tetramethyl-, [1aR-

			(1a.alpha.,1b.beta.,1c.beta.,2a.beta.,3a.alpha.,6a.alpha.,6b.alpha.,7.alpha.,8.beta.,8a.alpha.)]-
20	27.298	0.46	4H-Cyclopropa[5',6']benz[1',2':7,8]azuleno[5,6-b]oxiren-4-one, 8-(acetyloxy)-1,1a,1b,1c,2a,3,3a,6a,6b,7,8,8a-dodecahydro-3a,6b,8a-trihydroxy-2a-(hydroxymethyl)-1,1,5,7-tetramethyl-, (1a.alpha.,1b.beta.,1c.beta.,2a.beta.,3a.beta.,6a.alpha.,6b.alpha.,7.alpha.,8.beta.,8a.alpha.)-
21	27.917	1.68	4H-Cyclopropa[5',6']benz[1',2':7,8]azuleno[5,6-b]oxiren-4-one, 8-(acetyloxy)-1,1a,1b,1c,2a,3,3a,6a,6b,7,8,8a-dodecahydro-3a,6b,8a-trihydroxy-2a-(hydroxymethyl)-1,1,5,7-tetramethyl-, (1a.alpha.,1b.beta.,1c.beta.,2a.beta.,3a.beta.,6a.alpha.,6b.alpha.,7.alpha.,8.beta.,8a.alpha.)-
22	28.423	3.57	4H-Cyclopropa[5',6']benz[1',2':7,8]azuleno[5,6-b]oxiren-4-one, 8-(acetyloxy)-1,1a,1b,1c,2a,3,3a,6a,6b,7,8,8a-dodecahydro-3a,6b,8a-trihydroxy-2a-(hydroxymethyl)-1,1,5,7-tetramethyl-, (1a.alpha.,1b.beta.,1c.beta.,2a.beta.,3a.beta.,6a.alpha.,6b.alpha.,7.alpha.,8.beta.,8a.alpha.)-
23	28.766	0.65	4H-Cyclopropa[5',6']benz[1',2':7,8]azuleno[5,6]oxiren-4-one, 8,8a-bis(acetyloxy)-2a-[(acetyloxy)methyl]-1,1a,1b,1c,2a,3,3a,6a,6b,7,8,8a-dodecahydro-6b-hydroxy-3a-methoxy-1,1,5,7-tetramethyl-, [1aR-(1a.alpha.,1b.beta.,1c.beta.,2a.beta.,3a.alpha.,6a.alpha.,6b.alpha.,7.alpha.,8.beta.,8a.alpha.)]-
24	28.812	0.46	4H-Cyclopropa[5',6']benz[1',2':7,8]azuleno[5,6]oxiren-4-one, 8,8a-bis(acetyloxy)-2a-[(acetyloxy)methyl]-1,1a,1b,1c,2a,3,3a,6a,6b,7,8,8a-dodecahydro-6b-hydroxy-3a-methoxy-1,1,5,7-tetramethyl-, [1aR-(1a.alpha.,1b.beta.,1c.beta.,2a.beta.,3a.alpha.,6a.alpha.,6b.alpha.,7.alpha.,8.beta.,8a.alpha.)]-
25	29.051	11.90	2,6-Bis(3,4-methylenedioxyphenyl)-3,7-dioxabicyclo(3.3.0)octane
26	29.245	0.71	4H-Cyclopropa[5',6']benz[1',2':7,8]azuleno[5,6]oxiren-4-one, 8,8a-bis(acetyloxy)-2a-[(acetyloxy)methyl]-1,1a,1b,1c,2a,3,3a,6a,6b,7,8,8a-dodecahydro-6b-hydroxy-3a-methoxy-1,1,5,7-tetramethyl-, [1aR-

			(1a.alpha.,1b.beta.,1c.beta.,2a.beta.,3a.alpha.,6a.alpha.,6b.alpha.,7.alpha.,8.beta.,8a.alpha.)]-
27	29.387	1.41	4H-Cyclopropa[5',6']benz[1',2':7,8]azuleno[5,6]oxiren-4-one, 8,8a-bis(acetyloxy)-2a-[(acetyloxy)methyl]-1,1a,1b,1c,2a,3,3a,6a,6b,7,8,8a-dodecahydro-6b-hydroxy-3a-methoxy-1,1,5,7-tetramethyl-, [1aR-(1a.alpha.,1b.beta.,1c.beta.,2a.beta.,3a.alpha.,6a.alpha.,6b.alpha.,7.alpha.,8.beta.,8a.alpha.)]-
28	29.568	0.66	4H-Cyclopropa[5',6']benz[1',2':7,8]azuleno[5,6]oxiren-4-one, 8,8a-bis(acetyloxy)-2a-[(acetyloxy)methyl]-1,1a,1b,1c,2a,3,3a,6a,6b,7,8,8a-dodecahydro-6b-hydroxy-3a-methoxy-1,1,5,7-tetramethyl-, [1aR-(1a.alpha.,1b.beta.,1c.beta.,2a.beta.,3a.alpha.,6a.alpha.,6b.alpha.,7.alpha.,8.beta.,8a.alpha.)]-
29	30.403	7.93	4H-Cyclopropa[5',6']benz[1',2':7,8]azuleno[5,6-b]oxiren-4-one, 8-(acetyloxy)-1,1a,1b,1c,2a,3,3a,6a,6b,7,8,8a-dodecahydro-3a,6b,8a-trihydroxy-2a-(hydroxymethyl)-1,1,5,7-tetramethyl-, (1a.alpha.,1b.beta.,1c.beta.,2a.beta.,3a.beta.,6a.alpha.,6b.alpha.,7.alpha.,8.beta.,8a.alpha.)-
30	30.474	3.55	4H-Cyclopropa[5',6']benz[1',2':7,8]azuleno[5,6-b]oxiren-4-one, 8-(acetyloxy)-1,1a,1b,1c,2a,3,3a,6a,6b,7,8,8a-dodecahydro-3a,6b,8a-trihydroxy-2a-(hydroxymethyl)-1,1,5,7-tetramethyl-, (1a.alpha.,1b.beta.,1c.beta.,2a.beta.,3a.beta.,6a.alpha.,6b.alpha.,7.alpha.,8.beta.,8a.alpha.)-
31	30.539	4.42	4H-Cyclopropa[5',6']benz[1',2':7,8]azuleno[5,6-b]oxiren-4-one, 8-(acetyloxy)-1,1a,1b,1c,2a,3,3a,6a,6b,7,8,8a-dodecahydro-3a,6b,8a-trihydroxy-2a-(hydroxymethyl)-1,1,5,7-tetramethyl-, (1a.alpha.,1b.beta.,1c.beta.,2a.beta.,3a.beta.,6a.alpha.,6b.alpha.,7.alpha.,8.beta.,8a.alpha.)-
32	30.707	2.91	4H-Cyclopropa[5',6']benz[1',2':7,8]azuleno[5,6]oxiren-4-one, 8,8a-bis(acetyloxy)-2a-[(acetyloxy)methyl]-1,1a,1b,1c,2a,3,3a,6a,6b,7,8,8a-dodecahydro-6b-hydroxy-3a-methoxy-1,1,5,7-tetramethyl-, [1aR-(1a.alpha.,1b.beta.,1c.beta.,2a.beta.,3a.alpha.,6a.alpha.,6b.alpha.,7.alpha.,8.beta.,8a.alpha.)]-

Table S6. GC-MS analysis of bark ethanol/benzene extract**.**

No.	Retention Time (min)	Peak Area (%)	Component
1	4.85	11.55	Formamide, N,N-diethyl-
2	6.29	9.98	1-Hexanol, 2-ethyl-
3	10.88	0.45	5H-1-Pyrindine
4	18.55	0.54	Acetamide, N-methyl-N-[4-(3-hydroxypyrrolidinyl)-2-butynyl]-
5	19.96	3.78	Hexadecanoic acid, 2-methyl-
6	20.38	1.60	i-Propyl 14-methyl-pentadecanoate
7	20.77	0.58	1H-2,8a-Methanocyclopenta[a]cyclopropa[e]cyclodecen-11-one, 1a,2,5,5a,6,9,10,10a-octahydro-5,5a,6-trihydroxy-1,4-bis(hydroxymethyl)-1,7,9-trimethyl-, [1S-(1.alpha.,1a.alpha.,2.alpha.,5.beta.,5a.beta.,6.beta.,8a.alpha.,9.alpha.,10a.alpha.)]-
8	22.04	2.50	11-Octadecenoic acid, methyl ester
9	22.32	3.12	Heptadecanoic acid, 15-methyl-, methyl ester
10	22.44	3.99	(Z)-18-Octadec-9-enolide
11	22.50	2.71	1-Heptatriacotanol
12	22.61	0.49	1H-2,8a-Methanocyclopenta[a]cyclopropa[e]cyclodecen-11-one, 1a,2,5,5a,6,9,10,10a-octahydro-5,5a,6-trihydroxy-1,4-bis(hydroxymethyl)-1,7,9-trimethyl-, [1S-(1.alpha.,1a.alpha.,2.alpha.,5.beta.,5a.beta.,6.beta.,8a.alpha.,9.alpha.,10a.alpha.)]-
13	22.73	1.99	1H-2,8a-Methanocyclopenta[a]cyclopropa[e]cyclodecen-11-one, 1a,2,5,5a,6,9,10,10a-octahydro-5,5a,6-trihydroxy-1,4-bis(hydroxymethyl)-1,7,9-trimethyl-, [1S-(1.alpha.,1a.alpha.,2.alpha.,5.beta.,5a.beta.,6.beta.,8a.alpha.,9.alpha.,10a.alpha.)]-
14	22.80	0.96	1H-2,8a-Methanocyclopenta[a]cyclopropa[e]cyclodecen-11-one, 1a,2,5,5a,6,9,10,10a-octahydro-5,5a,6-trihydroxy-1,4-bis(hydroxymethyl)-1,7,9-trimethyl-, [1S-(1.alpha.,1a.alpha.,2.alpha.,5.beta.,5a.beta.,6.beta.,8a.alpha.,9.alpha.,10a.alpha.)]-
15	24.01	0.64	Ethyl stearate, mono 9-epoxy
16	24.79	0.60	1b,4a-Epoxy-2H-

			cyclopenta[3,4]cyclopropa[8,9]cycloundec[1,2-b]oxiren-5(6H)-one, 7-(acetyloxy)decahydro-2,9,10-trihydroxy-3,6,8,8,10a-pentamethyl-
17	26.08	10.26	1,22-Docosanediol
18	26.17	0.76	17-Pentatriacontene
19	26.25	0.47	.beta.-Sitosterol
20	26.46	6.63	.beta.-Sitosterol
21	26.96	1.16	1H-Cyclopropa[3,4]benz[1,2-e]azulene-5,7b,9,9a-tetrol, 1a,1b,4,4a,5,7a,8,9-octahydro-3-(hydroxymethyl)-1,1,6,8-tetramethyl-, 5,9,9a-triacetate, [1aR-(1a.alpha.,1b.beta.,4a.beta.,5.beta.,7a.alpha.,7b.alpha.,8.alpha.,9.beta.,9a.alpha.)]-
22	27.16	0.64	16-Allopregnen-3.beta.-ol-20-one
23	27.23	1.63	1H-Cyclopropa[3,4]benz[1,2-e]azulene-4a,5,7b,9,9a(1aH)-pentol, 1b,4,5,7a,8,9-hexahydro-3-(hydroxymethyl)-1,1,6,8-tetramethyl-, 9,9a-diacetate, [1aR-(1a.alpha.,1b.beta.,4a.beta.,5.beta.,7a.alpha.,7b.alpha.,8.alpha.,9.beta.,9a.alpha.)]-
24	27.90	2.43	17-Pentatriacontene
25	28.38	2.51	1H-Cyclopropa[3,4]benz[1,2-e]azulene-4a,5,7b,9,9a(1aH)-pentol, 1b,4,5,7a,8,9-hexahydro-3-(hydroxymethyl)-1,1,6,8-tetramethyl-, 9,9a-diacetate, [1aR-(1a.alpha.,1b.beta.,4a.beta.,5.beta.,7a.alpha.,7b.alpha.,8.alpha.,9.beta.,9a.alpha.)]-
26	28.63	0.45	1H-Cyclopropa[3,4]benz[1,2-e]azulene-4a,5,7b,9,9a(1aH)-pentol, 1b,4,5,7a,8,9-hexahydro-3-(hydroxymethyl)-1,1,6,8-tetramethyl-, 9,9a-diacetate, [1aR-(1a.alpha.,1b.beta.,4a.beta.,5.beta.,7a.alpha.,7b.alpha.,8.alpha.,9.beta.,9a.alpha.)]-
27	28.95	6.78	(+)-Sesamin
28	29.21	0.62	1H-Cyclopropa[3,4]benz[1,2-e]azulene-5,7b,9,9a-tetrol, 1a,1b,4,4a,5,7a,8,9-octahydro-3-(hydroxymethyl)-1,1,6,8-tetramethyl-, 5,9,9a-triacetate, [1aR-(1a.alpha.,1b.beta.,4a.beta.,5.beta.,7a.alpha.,7b.alpha.,8.alpha.,9.beta.,9a.alpha.)]-
29	29.29	1.07	1H-Cyclopropa[3,4]benz[1,2-e]azulene-5,7b,9,9a-tetrol, 1a,1b,4,4a,5,7a,8,9-octahydro-3-(hydroxymethyl)-1,1,6,8-tetramethyl-, 5,9,9a-triacetate, [1aR-

			(1a.alpha.,1b.beta.,4a.beta.,5.beta.,7a.alpha.,7b.alpha.,8.alpha.,9.beta.,9a.alpha.)]-
30	29.52	0.95	1H-Cyclopropa[3,4]benz[1,2-e]azulene-4a,5,7b,9,9a(1aH)-pentol, 1b,4,5,7a,8,9-hexahydro-3-(hydroxymethyl)-1,1,6,8-tetramethyl-, 9,9a-diacetate, [1aR-(1a.alpha.,1b.beta.,4a.beta.,5.beta.,7a.alpha.,7b.alpha.,8.alpha.,9.beta.,9a.alpha.)]-
31	30.10	14.72	17-(1,5-Dimethyl-3-phenylthiohex-4-enyl)-4,4,10,13,14-pentamethyl-2,3,4,5,6,7,10,11,12,13,14,15,16,17-tetradecahydro-1H-cyclopent(a)phenanthren-3-ol
32	30.56	1.43	17-(2-Hydroxy-1,5-dimethyl-hex-4-enyl)-4,4,10,13,14-pentamethyl-2,3,4,5,6,7,10,11,12,13,14,15,16,17-tetradecahydro-1H-cyclopenta[a]phenanthrene
33	30.68	1.99	1H-Cyclopropa[3,4]benz[1,2-e]azulene-4a,5,7b,9,9a(1aH)-pentol, 1b,4,5,7a,8,9-hexahydro-3-(hydroxymethyl)-1,1,6,8-tetramethyl-, 9,9a-diacetate, [1aR-(1a.alpha.,1b.beta.,4a.beta.,5.beta.,7a.alpha.,7b.alpha.,8.alpha.,9.beta.,9a.alpha.)]-

Table S7. GC-MS analysis of bark ethanol/methanol extract**.**

No.	Retention Time (min)	Peak Area (%)	Component
1	4.75	2.70	Formamide, N,N-diethyl-
2	6.20	2.47	1-Hexanol, 2-ethyl-
3	10.89	1.67	Indole
4	13.93	0.85	d-Mannose
5	17.61	0.61	(E)-4-(3-Hydroxyprop-1-en-1-yl)-2-methoxyphenol
6	18.58	0.76	Acetamide, N-methyl-N-[4-(3-hydroxypyrrolidinyl)-2-butynyl]-
7	19.96	5.10	Hexadecanoic acid, methyl ester
8	20.38	2.83	n-Hexadecanoic acid
9	20.78	0.63	1H-2,8a-Methanocyclopenta[a]cyclopropa[e]cyclodecen-11-one, 1a,2,5,5a,6,9,10,10a-octahydro-5,5a,6-trihydroxy-1,4-bis(hydroxymethyl)-1,7,9-trimethyl-, [1S-(1.alpha.,1a.alpha.,2.alpha.,5.beta.,5a.beta.,6.beta.,8a.alpha.,9.alpha.,10a.alpha.)]-
10	22.04	3.37	9-Octadecenoic acid, methyl ester, (E)-
11	22.32	4.71	Methyl stearate

12	22.41	5.60	9,12-Octadecadienoic acid (Z,Z)-
13	22.47	5.22	1-Heptatriacotanol
14	22.60	0.97	4H-Cyclopropa[5',6']benz[1',2':7,8]azuleno[5,6-b]oxiren-4-one, 8-(acetyloxy)-1,1a,1b,1c,2a,3,3a,6a,6b,7,8,8a-dodecahydro-3a,6b,8a-trihydroxy-2a-(hydroxymethyl)-1,1,5,7-tetramethyl-, [1ar-(1a.alpha.,1b.beta.,1c.alpha.,2a.alpha.,3a.beta.,6a.alpha.,6b.alpha.,7.alpha.,8.beta.,8a.alpha.)]-
15	22.70	2.45	4H-Cyclopropa[5',6']benz[1',2':7,8]azuleno[5,6-b]oxiren-4-one, 8-(acetyloxy)-1,1a,1b,1c,2a,3,3a,6a,6b,7,8,8a-dodecahydro-3a,6b,8a-trihydroxy-2a-(hydroxymethyl)-1,1,5,7-tetramethyl-, [1ar-(1a.alpha.,1b.beta.,1c.alpha.,2a.alpha.,3a.beta.,6a.alpha.,6b.alpha.,7.alpha.,8.beta.,8a.alpha.)]-
16	22.79	1.42	4H-Cyclopropa[5',6']benz[1',2':7,8]azuleno[5,6-b]oxiren-4-one, 8-(acetyloxy)-1,1a,1b,1c,2a,3,3a,6a,6b,7,8,8a-dodecahydro-3a,6b,8a-trihydroxy-2a-(hydroxymethyl)-1,1,5,7-tetramethyl-, (1a.alpha.,1b.beta.,1c.beta.,2a.beta.,3a.beta.,6a.alpha.,6b.alpha.,7.alpha.,8.beta.,8a.alpha.)-
17	22.94	0.61	4H-Cyclopropa[5',6']benz[1',2':7,8]azuleno[5,6-b]oxiren-4-one, 8-(acetyloxy)-1,1a,1b,1c,2a,3,3a,6a,6b,7,8,8a-dodecahydro-3a,6b,8a-trihydroxy-2a-(hydroxymethyl)-1,1,5,7-tetramethyl-, (1a.alpha.,1b.beta.,1c.beta.,2a.beta.,3a.beta.,6a.alpha.,6b.alpha.,7.alpha.,8.beta.,8a.alpha.)-
18	24.02	0.62	1H-Cyclopropa[3,4]benz[1,2-e]azulene-5,7b,9,9a-tetrol, 1a,1b,4,4a,5,7a,8,9-octahydro-3-(hydroxymethyl)-1,1,6,8-tetramethyl-, 5,9,9a-triacetate, [1aR-(1a.alpha.,1b.beta.,4a.beta.,5.beta.,7a.alpha.,7b.alpha.,8.alpha.,9.beta.,9a.alpha.)]-
19	24.79	0.58	1H-Cyclopropa[3,4]benz[1,2-e]azulene-5,7b,9,9a-tetrol, 1a,1b,4,4a,5,7a,8,9-octahydro-3-(hydroxymethyl)-1,1,6,8-tetramethyl-, 5,9,9a-triacetate, [1aR-(1a.alpha.,1b.beta.,4a.beta.,5.beta.,7a.alpha.,7b.alpha.,8.alpha.,9.beta.,9a.alpha.)]-
20	25.97	7.75	8,14-Seco-3,19-epoxyandrostane-8,14-dione, 17-acetoxy-3.beta.-methoxy-4,4-dimethyl-

21	26.18	1.20	4H-Cyclopropa[5',6']benz[1',2':7,8]azuleno[5,6-b]oxiren-4-one, 8-(acetyloxy)-1,1a,1b,1c,2a,3,3a,6a,6b,7,8,8a-dodecahydro-3a,6b,8a-trihydroxy-2a-(hydroxymethyl)-1,1,5,7-tetramethyl-, (1a.alpha.,1b.beta.,1c.beta.,2a.beta.,3a.beta.,6a.alpha.,6b.alpha.,7.alpha.,8.beta.,8a.alpha.)-
22	26.46	14.40	.gamma.-Sitosterol
23	26.97	0.97	4H-Cyclopropa[5',6']benz[1',2':7,8]azuleno[5,6]oxiren-4-one, 8,8a-bis(acetyloxy)-2a-[(acetyloxy)methyl]-1,1a,1b,1c,2a,3,3a,6a,6b,7,8,8a-dodecahydro-6b-hydroxy-3a-methoxy-1,1,5,7-tetramethyl-, [1aR-(1a.alpha.,1b.beta.,1c.beta.,2a.beta.,3a.alpha.,6a.alpha.,6b.alpha.,7.alpha.,8.beta.,8a.alpha.)]-
24	27.25	1.14	4H-Cyclopropa[5',6']benz[1',2':7,8]azuleno[5,6-b]oxiren-4-one, 8-(acetyloxy)-1,1a,1b,1c,2a,3,3a,6a,6b,7,8,8a-dodecahydro-3a,6b,8a-trihydroxy-2a-(hydroxymethyl)-1,1,5,7-tetramethyl-, (1a.alpha.,1b.beta.,1c.beta.,2a.beta.,3a.beta.,6a.alpha.,6b.alpha.,7.alpha.,8.beta.,8a.alpha.)-
25	27.89	1.89	7,8-Epoxylanostan-11-ol, 3-acetoxy-
26	28.03	2.28	7,8-Epoxylanostan-11-ol, 3-acetoxy-
27	28.39	1.48	7,8-Epoxylanostan-11-ol, 3-acetoxy-
28	28.64	0.58	7,8-Epoxylanostan-11-ol, 3-acetoxy-
29	28.92	11.25	2,6-Bis(3,4-methylenedioxyphenyl)-3,7-dioxabicyclo(3.3.0)octane
30	29.21	0.83	4H-Cyclopropa[5',6']benz[1',2':7,8]azuleno[5,6]oxiren-4-one, 8,8a-bis(acetyloxy)-2a-[(acetyloxy)methyl]-1,1a,1b,1c,2a,3,3a,6a,6b,7,8,8a-dodecahydro-6b-hydroxy-3a-methoxy-1,1,5,7-tetramethyl-, [1aR-(1a.alpha.,1b.beta.,1c.beta.,2a.beta.,3a.alpha.,6a.alpha.,6b.alpha.,7.alpha.,8.beta.,8a.alpha.)]-
31	29.31	2.86	4H-Cyclopropa[5',6']benz[1',2':7,8]azuleno[5,6-b]oxiren-4-one, 8-(acetyloxy)-1,1a,1b,1c,2a,3,3a,6a,6b,7,8,8a-dodecahydro-3a,6b,8a-trihydroxy-2a-(hydroxymethyl)-1,1,5,7-tetramethyl-, (1a.alpha.,1b.beta.,1c.beta.,2a.beta.,3a.beta.,6a.alpha.,6b.alpha.,7.alpha.,8.beta.,8a.alpha.)-
32	29.54	0.76	7,8-Epoxylanostan-11-ol, 3-acetoxy-
33	30.06	3.60	5H-Cyclopropa[3,4]benz[1,2-e]azulen-5-one, 9,9a-

			bis(acetyloxy)-3-[(acetyloxy)methyl]-2-chloro-1,1a,1b,2,3,4,4a,7a,7b,8,9,9a-dodecahydro-3,4a,7b-trihydroxy-1,1,6,8-tetramethyl-, [1aR-(1a.alpha.,1b.beta.,2.alpha.,3.alpha.,4a.beta.,7a.alpha.,7b.alpha.,8.alpha.,9.beta.,9a.alpha.)]-
34	30.11	2.84	5H-Cyclopropa[3,4]benz[1,2-e]azulen-5-one, 9,9a-bis(acetyloxy)-3-[(acetyloxy)methyl]-2-chloro-1,1a,1b,2,3,4,4a,7a,7b,8,9,9a-dodecahydro-3,4a,7b-trihydroxy-1,1,6,8-tetramethyl-, [1aR-(1a.alpha.,1b.beta.,2.alpha.,3.alpha.,4a.beta.,7a.alpha.,7b.alpha.,8.alpha.,9.beta.,9a.alpha.)]-
35	30.58	1.78	4H-Cyclopropa[5',6']benz[1',2':7,8]azuleno[5,6]oxiren-4-one, 8,8a-bis(acetyloxy)-2a-[(acetyloxy)methyl]-1,1a,1b,1c,2a,3,3a,6a,6b,7,8,8a-dodecahydro-6b-hydroxy-3a-methoxy-1,1,5,7-tetramethyl-, [1aR-(1a.alpha.,1b.beta.,1c.beta.,2a.beta.,3a.alpha.,6a.alpha.,6b.alpha.,7.alpha.,8.beta.,8a.alpha.)]-
36	30.69	1.22	7,8-Epoxylanostan-11-ol, 3-acetoxy-

Table S8. LC-QTOF-MS analysis of bark ethanol/methanol extract

No.	Retention Time (min)	Peak Area (%)	Component
1	0.5	0.147	Cappariloside B
2	0.5	0.026	Buxbodine C
3	0.5	0.026	(+)-Gomisin K2
4	0.5	0.265	Cappariloside B
5	0.5	0.039	Cibarian
6	0.5	0.016	Methyl 2,3,6-tri-O-galloyl-beta-D-glucopyranoside
7	0.5	0.050	Miltionone I
8	0.5	0.018	Aucubigenin
9	0.6	0.127	ent-8,9-Seco-7alpha-hydroxy-11-acetoxykaura-8(14),16-dien-9,15-dione
10	0.6	0.020	Adouetine Y
11	0.6	2.293	Echinopsine
12	0.6	0.089	Cyperaquinone
13	0.6	0.116	Phenethylamine
14	0.6	3.040	Betaine
15	0.6	0.017	19-(R)-Hydroxydihydrokoumine
16	0.7	0.022	Phyllanthin
17	0.7	0.041	16-Acetoxy-7alpha-methoxyroyleanone

18	0.8	5.623	Erycibelline
19	0.8	0.019	Acrifoline
20	0.8	24.682	Nb-Methyltetrahydroharman
21	0.8	16.918	Bufotenidine
22	0.9	0.020	Arenobufagin
23	0.9	0.018	Raunescine
24	0.9	0.698	Indole
25	0.9	0.017	Bufarenogin
26	0.9	0.044	16-Acetoxy-7alpha-methoxyroyleanone
27	0.9	0.020	Phyllanthin
28	1	0.016	Bufarenogin
29	1	0.054	Homoferreirin
30	1	0.030	Nauclefine
31	1	0.422	Holadysine
32	1.2	0.034	Nauclefine
33	1.2	0.019	Angustoline
34	1.2	1.095	Pegamine
35	1.2	0.073	Cinnamaldehyde
36	1.3	0.017	Homoferreirin
37	1.3	0.028	16-Epi-voacarpine
38	1.3	11.938	2-Methyl-6-methoxy-1,2,3,4-tetrahydro-beta-carboline
39	1.3	0.055	Echinopsine
40	1.4	0.118	gamma-Guanidinobutyric acid
41	1.4	0.037	3,4-Dimethyl-5-phenyloxazolidine
42	1.4	0.152	Indole
43	1.4	4.561	Patriscabratine
44	1.4	0.018	Knoxiadin
45	1.4	0.363	Nauclefidine
46	1.6	0.854	4-Methoxyl-1-methyl-2-quinolone
47	1.6	0.024	Rheumin
48	1.6	4.024	Hypaphorine
49	1.6	2.252	Corynantheine
50	1.7	0.729	(+)-2,3-Dehydro-10-oxo-alpha-isosparteine
51	1.8	0.039	Macrophyllin B
52	1.8	0.016	Brucine N-oxide
53	1.8	0.053	16-Methoxycarbonyl naufoline
54	1.8	0.015	Annotinine
55	1.8	0.073	Creatinine
56	1.8	0.026	Deoxyvasicinone

57	2.1	0.128	Anonaine
58	2.1	0.421	Phthalic anhydride
59	2.2	0.062	Gentiatibetine
60	2.2	0.028	Acrifoline
61	2.2	0.048	Indole
62	2.3	0.015	Raunescine
63	2.3	0.018	Ammiol
64	2.3	0.024	Holadysamine
65	2.3	0.034	Patriscabratine
66	2.3	0.035	gamma-Camphorene
67	2.5	0.016	(+)-Fustin
68	2.6	0.053	2-Methyl-3-(2',3',4'-trihydroxybutyl)pyrazine
69	2.7	0.033	1-Carboethoxy-beta-carboline
70	2.7	0.046	N-Desmethoxyrankinidine
71	2.7	0.030	19-(R)-Hydroxydihydrokoumine
72	2.8	0.074	Annuionone E
73	2.8	0.198	Alloyohimbine
74	2.8	0.015	Gerronemin B
75	2.8	0.036	Anatabine
76	2.8	0.120	Patriscabratine
77	2.9	0.186	Triricinolein
78	2.9	0.014	Isogentisin
79	3	0.058	Homoferreirin
80	3	0.050	1-Carboethoxy-beta-carboline
81	3.2	1.229	Patriscabratine
82	3.2	0.043	Dictysine
83	3.2	0.140	6-Methyl indole
84	3.2	0.013	Aurantiamide acetate
85	3.3	0.433	L-Epigallocatechin
86	3.3	1.029	Epicatechin
87	3.3	0.033	Gentianine
88	3.3	0.360	6-Methyl indole
89	3.4	0.075	Bujeine
90	3.4	0.035	10-Hydroxyangustine
91	3.4	0.068	Alloisoleucine
92	3.4	0.157	Dictafolin B
93	3.4	0.026	Prenyl caffeate
94	3.4	0.029	Anthraquinone
95	3.5	0.061	(+)-Fustin
96	3.5	0.273	Diisocapryl phthalate

97	3.5	0.192	L-Epigallocatechin
98	3.6	0.023	Rosacea acid A
99	3.6	0.096	Acronycine
100	3.6	0.023	6-Hydroxyluteolin
101	3.8	0.152	Eleutherol
102	3.8	0.065	Loliolide
103	3.8	0.201	Alstovenine
104	3.9	0.019	6,7-Dimethoxy-2-[2-(4'-methoxyphenyl)ethyl]chromone
105	4	0.271	5-Methoxy-N,N-dimethyl-tryptamine Nb-oxide
106	4	0.271	Alloyohimbine
107	4	0.014	N-Desmethoxyrankinidine
108	4	0.021	Caribine
109	4.1	0.043	N-Demethyl-acronycine
110	4.1	0.041	alpha-Hydroxyanthraquinone
111	4.2	0.033	Chinensinaphthol
112	4.2	0.308	10-Hydroxyangustine
113	4.2	0.030	(+)-Fustin
114	4.2	0.033	Aucubigenin
115	4.2	0.732	19-(R)-Hydroxydihydrokoumine
116	4.2	0.026	Aromadendrin-5,7-dimethyl ether
117	4.2	0.031	alpha-Hydrojuglone
118	4.2	0.013	Przewaquinone F
119	4.3	0.034	(+)-Nb-Methyl tryptophan methyl ester (S)
120	4.4	0.237	2-Methyl-6-methoxy-1,2,3,4-tetrahydro-beta-carboline
121	4.4	0.063	Daidzein 4',7-diglucoside
122	4.4	0.207	Abrine
123	4.4	0.036	Vitamin B5
124	4.4	0.130	16-Epi-voacarpine
125	4.4	0.231	Harmol
126	4.4	0.028	Methyl isopelletierine
127	4.4	0.064	Alloisoleucine
128	4.4	0.020	Bujeine
129	4.4	0.025	(-)-Eudesm-3-ene-6alpha-acetoxy-7alpha-ol
130	4.5	0.015	Angustine
131	4.5	0.031	Japonine
132	4.5	0.033	Hiyodorilactone B
133	4.5	0.064	Patriscabratine
134	4.5	0.136	14-Fomyldihydroxyrutaecarpine

135	4.5	0.020	Alstovenine
136	4.5	0.050	Biondnoid I
137	4.5	0.042	16-Epi-voacarpine
138	4.6	0.108	Xanthine
139	4.6	0.135	Harmine
140	4.6	0.033	Corynantheine
141	4.6	0.083	Rankinidine
142	4.7	0.065	5,6-Dimethyl-3a,4,7,7a-tetrahydro-1,3-lsoben-zofurandione
143	4.7	0.015	Taraxinic acid 1'-O-beta-D-glucopyranoside
144	4.7	0.032	4,8-Dimethoxy-1-(2-methoxyethyl)-beta-carboline
145	4.7	0.023	Biondnoid I
146	4.8	0.458	Corynantheine
147	4.8	0.157	Angustine
148	4.8	0.455	Epicatechin
149	4.8	0.022	Armillaripin
150	4.8	0.061	(+)-Nb-Methyl tryptophan methyl ester (S)
151	4.8	0.014	Raunescine
152	4.8	0.018	Berneuxia saponin C
153	4.9	0.048	Guvacine
154	4.9	0.015	Bujeine
155	5	0.050	(E,E,E)-N-(2-Methylpropyl)-hexadeca-2,6,8-trien-10-ynamide
156	5	0.100	7-Hydroxy-3-butylidene-phthalide
157	5	0.018	Aurantiamide acetate
158	5.1	0.053	N-Demethyl-acronycine
159	5.1	0.126	gamma-Amino-alpha-methylene butyric acid
160	5.2	0.141	Angustine
161	5.2	0.034	Desmosflavone
162	5.3	0.018	Dentatin
163	5.4	0.102	12-Methoxy-Na-methyl-vellosimine
164	5.4	0.023	Desmosflavone
165	5.4	0.016	Dianoside G
166	5.4	0.133	Aurantiamide acetate
167	5.4	0.040	(2E,4E,8E,10E,12E)-N-Isobutyl-2,4,8,10,12-tetradecapentaenamide
168	5.4	0.022	10-O-Acetylmonotropein
169	5.5	0.020	Karounidiol
170	5.5	0.069	Fluorocarpamine-N-oxide
171	5.5	0.326	(E,E,E)-N-(2-Methylpropyl)-hexadeca-2,6,8-trien-10-ynamide

172	5.5	0.025	Angustine
173	5.6	0.051	Abrine
174	5.6	0.017	Dihydrocorynantheine
175	5.6	0.041	Biondnoid I
176	5.6	0.072	19-O-Methylangustoline
177	5.7	0.074	Angustine
178	5.7	0.046	2'-Methoxy-3,4,4'-trihydroxychalcone
179	5.7	0.041	Rotundone
180	5.7	0.020	Japonine
181	5.8	0.033	3-O-beta-D-Glucopyranosyl-(1-->6)-beta-D-glucopyranosyl oleanolic acid 28-beta-D-glucopyranosyl-(1--
182	5.8	0.080	Swainsonine
183	5.8	0.023	6-O-Acetylstritosamide
184	5.9	0.026	5-Hydroxy-7,2',6'-trimethoxyflavone
185	5.9	0.028	Ergoflavine
186	5.9	0.430	Norharman
187	6	0.022	12-Methoxy-Na-methyl-vellosimine
188	6	0.112	Dihydrorobinetin
189	6	0.026	Procyanidin C
190	6	0.021	Valepotriate
191	6	0.021	Cassigerol E
192	6.1	0.031	Procyanidin C
193	6.1	0.052	Neohopadiene
194	6.2	0.106	Brachystamide D
195	6.2	0.079	2-Methyl-6-methoxy-1,2,3,4-tetrahydro-beta-carboline
196	6.3	0.057	Rhododendrin
197	6.3	0.150	1-Acetyl-beta-carboline
198	6.4	0.029	Aromadendrin-5,7-dimethyl ether
199	6.4	0.023	4,8-Dimethoxy-1-(2-methoxyethyl)-beta-carboline
200	6.5	0.088	Adenosine
201	7	0.073	Prenyl caffeate
202	7.2	0.019	Procyanidin C
203	7.3	0.015	Lucidin
204	7.4	0.026	Bujeine
205	7.5	0.264	Harman
206	7.8	0.155	19-O-Methylangustoline
207	8.1	0.061	2,3-Dihydro-5,7-dihydroxy-2,6,8-trimethyl-4H-1-benzopyran-4-one
208	8.2	0.038	Gomaline

209	8.5	0.042	Heraclenol
210	8.6	0.022	Ergoflavine
211	8.9	0.162	1-Formyl-4-methoxy-beta-carboline
212	9.6	0.093	1-Formyl-4-methoxy-beta-carboline
213	9.6	0.086	(2E,4E,8E,10E,12E)-N-Isobutyl-2,4,8,10,12-tetradecapentaenamide
214	9.6	0.037	Physovenine
215	9.7	0.013	Methl butyric acid tussilagin ester
216	9.9	0.019	Corynantheine
217	9.9	0.032	Rotundone
218	10	0.057	Koumine N-oxide
219	10.2	0.019	Aurantiamide acetate
220	10.3	0.028	Leucodelphinidin
221	10.5	0.020	Bulbocapnine
222	10.6	0.071	2,3,4-Trimethyl-5-phenyloxazolidine
223	11	0.025	1-Methyl-2-dodecyl-4-(1H)- quinolone
224	11.8	0.081	Cyclo Ala-Ala
225	12.9	0.043	Gramine
226	13	0.032	10-Amino-2,4-dimethoxyphenanthrene-1-carboxylic acid lactam
227	13.5	0.032	Angustine
228	13.9	0.193	Methl butyric acid tussilagin ester
229	14	0.081	5-Methoxymethyl furfural
230	14.2	0.018	Vineridine
231	14.3	0.056	Adenine
232	14.5	0.014	Aromadendrin-5,7-dimethyl ether
233	15.8	0.085	Trichotomine
234	15.8	0.016	Corynantheine
235	15.8	0.063	Baptifoline
236	15.8	0.015	Campesteryl-D-glucoside-6'-palmitate
237	16.5	0.040	Trichotomine
238	18.1	0.049	Pentahydroxybufostane
239	19.6	0.072	Flavin mononucleotide
240	19.6	0.013	Alangimarine
241	20.1	0.051	alpha-Rotunol
242	20.5	0.016	Guvacine
243	20.6	0.069	Triptofordin B2
244	20.9	0.059	Bixin
245	27	0.047	Guvacine
246	27	0.031	Triptofordin B2

247	27.1	0.020	Mallotochromene
248	27.1	0.023	Triptofordin F2
249	27.1	0.036	Camelliagenin E
250	27.1	0.018	Dianoside C
251	27.1	0.015	Munjistin
252	27.1	0.022	(+)-Catechin-pentaacetate
253	27.1	0.015	3-O-Caffeoyl-4-O-sinapoylquinic acid
254	27.2	0.021	Aloeresin G
255	27.2	0.038	(2S)-5-Methoxy flavan-7-ol
256	27.3	0.987	Didrovaltratum
